# Drivers of the association between armed conflict and intimate partner violence: A systematic review

**DOI:** 10.1017/gmh.2026.10248

**Published:** 2026-06-15

**Authors:** Aine Travers, Victoria Flavia Namuggala, Ibrahim Bahati, Ciara Buckley

**Affiliations:** 1 School of Psychology, Dublin City University, Dublin, Ireland; 2 School of Women and Gender Studies, Makerere University, Kampala, Uganda; 3 Department of Geography and the Environment, The University of Texas at Austin, USA

**Keywords:** armed conflict, IPV, humanitarianism, intersectionality, PTSD

## Abstract

The association between armed conflict and intimate partner violence (IPV) is well established. However, the mechanisms or drivers of this relationship are less well understood. This review provides a systematic synthesis of published literature on the factors driving the association between violence in the public and private spheres. Five databases (Web of Science, EMBASE, CINAHL, PsycINFO and PubMed) were systematically searched to identify all studies examining potential drivers. Inclusion criteria specified that studies should be based on adult samples, should measure or analyse the impact of conflict exposure, and should provide some insight into the drivers of the association between armed conflict and IPV, rather than only documenting the association. A total of 49 studies (25 qualitative and 24 quantitative) met the inclusion criteria. Identified drivers included individual, relational and structural factors. Among the most empirically supported drivers were conflict-related trauma and post-traumatic stress disorder (PTSD), stress associated with the economic effects of conflict and changes to gender roles and norms in the post-conflict setting. The intersection of these factors, particularly gender roles and economic factors, also emerged as a significant dynamic across multiple studies. The findings highlight the importance of integrating gender considerations, including IPV prevention and response, into humanitarian programming. There is a need for further research and theory-building to better integrate the factors operating at both individual and societal levels, and to better incorporate consideration of the influence of historical factors such as legacies of imperialism and colonial violence.

## Impact statement

Violence in intimate relationships is more prevalent in communities affected by war and armed conflict. While this association is well documented, the mechanisms underlying it are less well understood. This review synthesises evidence on the individual, relational and structural drivers linking armed conflict to intimate partner violence (IPV). Key drivers include conflict-related trauma and PTSD, alcohol use, economic stress and insecurity and shifts in gender roles and norms, with evidence highlighting the intersection of gender and economic factors as a particularly significant dynamic. By identifying empirically supported drivers across multiple levels, this review provides evidence to inform prevention and response efforts in conflict-affected settings. The findings highlight the importance of integrating gender-transformative approaches and IPV prevention into humanitarian and post-conflict programming. However, the evidence base is disproportionately drawn from military samples in high-income countries, with comparatively limited research from low- and middle-income settings. Expanding research across diverse geographic and socio-political contexts is essential to developing a more comprehensive and globally relevant understanding of conflict-related IPV.

## Introduction

IPV encompasses physical, sexual or psychological violence that takes place between current or former intimate partners (WHO, 2024). This form of violence disproportionately affects women and presents a major threat to health and human rights worldwide. Several studies have documented the association between exposure to armed conflict and higher rates of IPV (e.g., Kelly et al., 2018; Spangaro et al., 2021; Bandara et al., 2022; Le and Nguyen, 2022). In their study of the intersections between armed conflict and IPV in Liberia, Kelly et al. (2018) identified a 50% increased risk of IPV associated with living in conflict-affected districts. This finding was replicated in a study in Sri Lanka (Bandara et al., 2022), which found that women residing in conflict-affected areas experienced significantly increased odds (OR 2.96) of experiencing IPV. A secondary analysis of Demographic and Health Survey (DHS) data drawn from across Africa (Le and Nguyen, 2022) further illustrated an association between the intensity of armed conflict and IPV experienced by women. A recent systematic review and meta-analysis (Murphy et al., 2025) found a pooled prevalence of 39% lifetime physical or sexual violence among women and girls in conflict-affected settings, and a 24% 12-month rate.

This evidence shows that after the cessation of public violence and the signing of peace agreements, severe violence often continues in the private sphere. Anticolonial theorists have long examined how colonial violence and destruction impact human psychology and relationships. In *The Wretched of the Earth*, Frantz Fanon (2001) described how French colonial violence in Algeria produced dehumanisation and aggression within colonised communities, sometimes surfacing in violence against partners and families. These analyses have been extended by feminist decolonial scholars (e.g., Lugones, 2007; Smith, 2015; Segato and Monque, 2021), who have further examined the ways in which colonial domination produces rigid gendered power structures that perpetuate gendered violence in post-colonial contexts.

The continuum of violence theory, first articulated by Kelly (1988), provides a framework for considering how everyday, often normalised acts of gendered harassment and coercion are connected to and reinforce more extreme forms of violence. Extending this analysis to war, peace and security, Cockburn et al. (2004) argued that gendered power relations normalise violence against women and girls, whereby militarism is directly connected to everyday violence against women and girls. Cockburn et al. (2004) demonstrated how this violence persists through displacement, economic and social reconstruction, and processes of aid, justice and reconciliation. True (2010) further showed how these dynamics are also shaped by structural political and economic forces. Violence in the domestic sphere constitutes an ongoing violation of rights in itself, and also threatens longer-term peace and stability. Such violence can perpetuate trauma-related social problems, an effect that can reverberate through generations in post-conflict settings (O’Neill et al., 2015). Domestic violence also prevents victims from participating in public life, which in turn impacts peace-building processes and reinforces inequality.

Traditionally, peacebuilding and humanitarian response efforts have often neglected to consider gender issues (Cockburn et al., 2004; True, 2010; Mulumba and Namuggala, 2014; Swaine, 2018), leading to calls to integrate gender considerations into humanitarian programming, including the Inter-Agency Standing Committee (IASC, 2015) ‘Guidelines for Integrating Gender-Based Violence Interventions in Humanitarian Action’, which sets out priorities for addressing gender-based violence in humanitarian emergencies. Such interventions must be supported by evidence, including an understanding of empirically supported risk factors.

The complex reasons for the greater prevalence of IPV in conflict-affected regions are increasingly being explored in a growing body of evidence (Ringdal, 2024; Murphy et al., 2025). Some drivers of the relationship that have been explored include rigid societal gender norms during times of conflict (Reilly et al., 2004; Ellsberg et al., 2020), conflict-related trauma (Bradley, 2018), stigma associated with conflict-related sexual violence (Annan and Brier, 2010); alcohol use (Kelly et al., 2018), breakdown of governance and rule of law (Pierson, 2019), disintegration of social systems and networks (Mannell et al., 2021a), and greater availability of weapons (Doyle and McWilliams, 2020). As explored by Judith Butler (2009) in *Frames of War*, social processes and communications that legitimise armed conflict cultivate a perception of war as inevitable or morally just, by defining which lives are grievable and which are not. This legitimisation of public violence in wartime may also translate to greater legitimisation of violence in the domestic sphere, as the ‘frame’ of legitimate violence expands.

However, understanding of the drivers of the relationship between domestic violence and armed conflict is a comparatively underdeveloped area in research. Studies of proposed drivers have tended to focus on the impact of war-related trauma (e.g., Hecker et al., 2015) and post-traumatic stress disorder (PTSD; e.g., Orcutt et al., 2003), with a focus on military samples. A review to broadly examine these and other potential drivers of the association is needed to better understand how to integrate prevention efforts into humanitarian programming and post-conflict peace-building. This review, therefore, aims to systematically synthesise evidence on the factors linking armed conflict and IPV.

## Methodology

### Search strategy

Five databases (Web of Science, EMBASE, CINAHL, PsycINFO and PubMed) were systematically searched using terms such as humanitarian, war, armed conflict, political violence, post-conflict, militant, refugee, displacement, cross-referenced with terms relating to IPV (e.g., partner violence, domestic violence, spousal abuse). The full search string is provided in Supplementary File 1.

**Figure 1.**
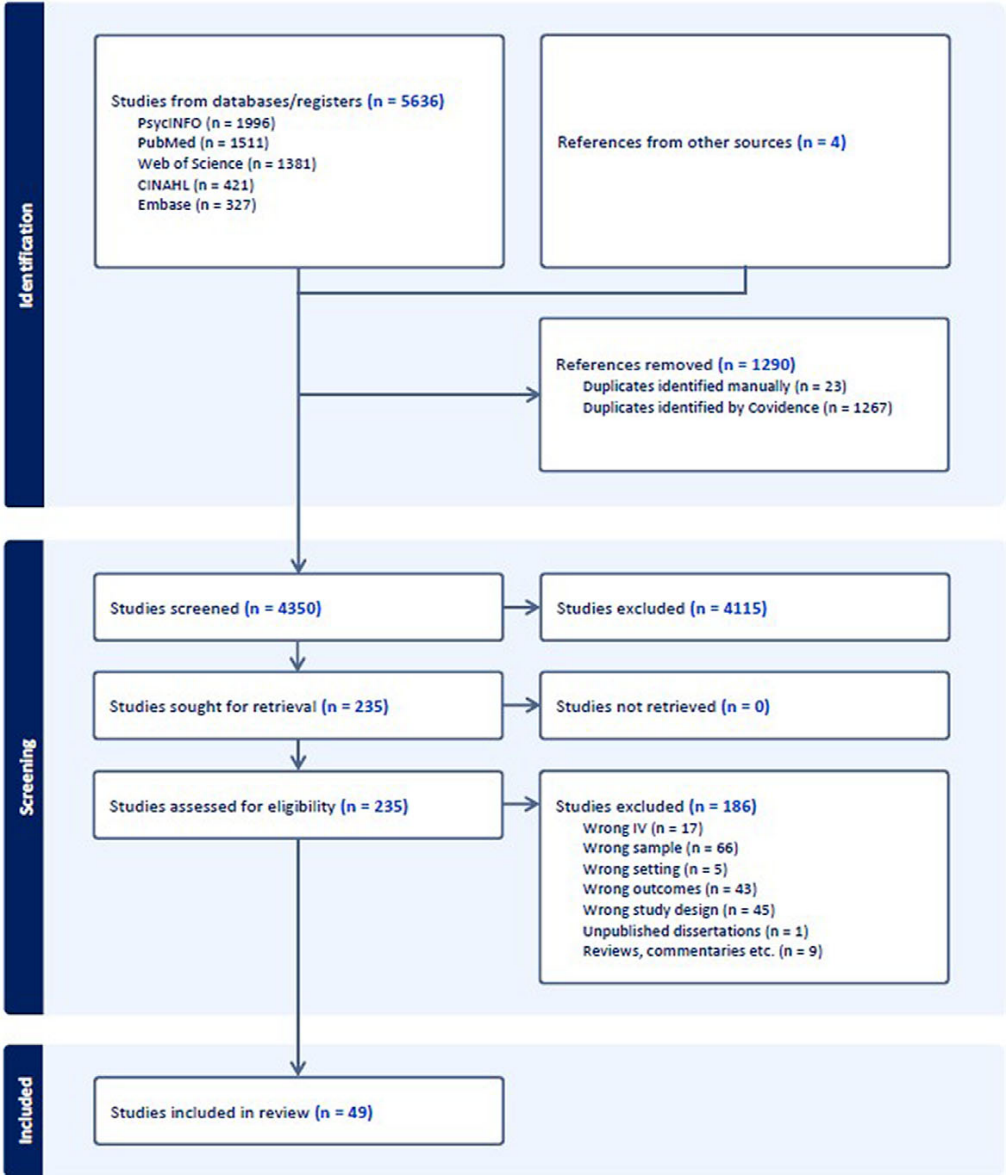
PRISMA diagram detailing screening process. Adapted from Page et al (2021). Figure 1. long description.

The initial search was conducted on 1 February 2024 and updated on 17 December 2025. There were 5,636 articles identified as potentially meeting the inclusion criteria. All references were exported to the systematic review platform Covidence. After removal of duplicates, 4,350 articles remained for title and abstract screening. All articles were dual-screened independently, first by title and abstract, and then in full text by two reviewers, with any conflicts in ratings settled through discussion with a third reviewer. Bibliographies of included articles were hand-searched for other potentially relevant articles. Four studies were identified through these means. See Figure 1 for further details of the screening procedure.

### Inclusion and exclusion criteria

Our inclusion criteria specified that studies should be based on adult (18+) samples of all genders, with exposure to armed conflict or political violence. Peer-reviewed quantitative, qualitative or mixed methods studies were included. It was required that studies either explicitly measured (for quantitative studies) or analysed (for qualitative studies) conflict exposure, meaning, for example, that studies with a general sample drawn from a conflict-affected region where it could not be ascertained whether participants had been exposed to armed conflict or violence were excluded. The outcome of interest was IPV, defined as physical, sexual or emotional abuse between current or former intimate partners. Any studies examining the association between armed conflict and other types of violence, such as family violence (e.g., violence against children, siblings or elders) and physical or sexual violence perpetrated by non-partners, were excluded. Studies were required to explore potential drivers of the association between armed conflict and IPV; therefore, studies only documenting the association between armed conflict and IPV without examining the factors linking them were excluded. Included studies were required to be primary research studies written in English; therefore, any reviews or commentary articles, as well as studies written in other languages, were excluded. No time restrictions were applied.

### Data extraction and analysis

Data were extracted using two pre-defined data extraction tables, developed collaboratively by the study team. Study information and setting were extracted, as well as sample details, study objectives, main findings and the potential driver identified. See Tables 1 and 2 for a summary of the study characteristics. Due to the methodological heterogeneity of studies included, a narrative synthesis was deemed the most appropriate approach to analysis. Study quality was assessed using the Mixed Methods Appraisal Tool (MMAT; Hong et al., 2018).

**Table 1.** Summary of qualitative studies included in the review Table 1. long description.

Authors, year, country	Sample details	Study design	Analysis	Study aims	Main findings	Proposed driver
Al-Natour et al. (2022), Jordan	Syrian refugee women (*n* = 16), aged 21–68 (*M* = 40). All experienced war, all married or divorced. All were Muslim, unemployed, *n* = 1 educated beyond the elementary level	Qualitative semi-structured interviews	Content analysis (Hsieh and Shannon, 2005)	To highlight the experiences of the Syrian war-refugee families who have sought shelter in a host country (Jordan)	War was a source of financial and social stress, sometimes resulting in family violence. War had wide-ranging adverse impacts on the health and well-being of women and children	Conflict-related unemployment led to financial stress, worsened by relocating to areas with a higher cost of living. Participants also reported that husbands’ behaviours and mental states changed as a result of war, with increased emotional difficulties contributing to increased IPV
Azizi et al. (2024), United Kingdom	Married Afghan women (*n* = 15) aged 27–60 (*M* = 36) seeking asylum or family reunification. One had graduated from university, the remainder had no formal training. At the time of the interviews, *n* = 1 participant was employed	Qualitative semi-structured interviews	Thematic analysis (Braun and Clarke, 2006)	To identify factors that facilitate or inhibit the integration of female Afghan refugees in London	Patriarchal power in Afghanistan operates at the state level and within families, resulting in violence against women pre- and post-migration. Lack of awareness of women’s rights increases dependence on male partners, heightening the risk of violence and difficulty integrating into a new environment	Additional vulnerabilities for victims may increase the risk of IPV. For example, in instances where couples have fled to other countries, perpetrators can take advantage of increased barriers to reporting for victims, including language barriers, lack of knowledge of authority systems and financial dependency on the perpetrator
Cardoso et al. (2016), Cote d’Ivoire	Ten focus groups (*N* = 91) of participants living in Abidjan, Cote d’Ivoire, aged 18 and above. Three groups of non-IDP women (*n* = 26), two IDP women (*n* = 20), three non-IDP men (*n* = 26) and two IDP men (*n* = 19)	Qualitative focus groups	Grounded theory	To understand the experiences of conflict-affected women living in urban areas and examine social and structural characteristics that contribute to IPV experiences among conflict-affected women	Loss of livelihoods and migration led to poverty, stress and reduced social support, which increased IPV. Changed gender roles, e.g., women’s increased economic independence, are perceived to increase risk. Discrimination increases vulnerability to poverty and IPV	Socio-economic and societal factors, including lack of employment opportunities, gender role changes and discrimination against IDP-identified people
Daoud (2021), Jordan	Syrian refugee women (*n* = 23) and service providers (*n* = 13). Women aged 18–42 (*M* = 30), married 4–24 years. Education: none (*n* = 5), primary (*n* = 15), secondary (*n* = 2), post-secondary (*n* = 1). Service providers aged 26–41 (*M* = 33). Bachelor’s degree (*n* = 8), master’s (*n* = 3), doctorate (*n* = 2)	Narrative interviews adhering to the guidelines of Ellsberg and Heise (2005)	Thematic analysis	To understand the conditions that increase risk of IPV among Syrian refugees in Jordan	Factors that create the risk of IPV are interconnected. Disruption of cultural codes; increased incidents of illegal marriages, conflict-related trauma and cultural norms which prevented survivors from reporting IPV incidents	Changing gender roles, e.g., men unable to provide for families, leading to shame, frustration, marital tension and IPV. Displaced women adopting a new culture perceived as threatening. Poverty, financial instability, unemployment, overcrowding, conflicts with others due to insufficient community resources, increased jealousy in new surroundings, conflict-related trauma all also identified as factors increasing risk, as well as stigma of wartime sexual violence
Doyle and McWilliams (2020), Northern Ireland	Two samples of women IPV survivors – from 1992 (*n* = 64), and from 2016 (*n* = 63). Of the 2016 sample, age groups were 18–29 (*n* = 9), 30–39 (*n* = 12), 40–49 (*n* = 19), 50–59 (*n* = 11), 60–69 (*n* = 11), 70+ (*n* = 1), 28 Catholic and 24 Protestant; remainder were mixed religion, Baptist, Methodist, Muslim or Other	Comparative qualitative interviews based on two samples gathered at different time points – one during conflict, the other during peacetime	Thematic analysis	To examine how the transition from violent conflict to peacetime affects women’s experiences of IPV	Themes of policing, paramilitaries and firearms. Many changes for IPV survivors came with the transition to peace, e.g., improved experience of reporting to police, less reliance on paramilitary groups for justice. However, some influence of the conflict persisted, e.g., perpetrators’ using paramilitary connections and access to firearms to exert control	Policing shapes the experience of IPV during conflict by affecting both the police responsiveness to violence and communities’ perceptions of the likelihood of police being helpful. Paramilitary membership and access to firearms create additional threats and opportunities for perpetrators to exert control over victims
Falb et al. (2014), Cote D’Ivoire	Adult men (*n* = 32) living in conflict-affected areas whose wives participated in a financial planning programme	Qualitative interviews	Inductive thematic analysis	To explore how men’s social identities manifest in a conflict-affected environment, their attitudes towards economic empowerment programming and reasons for participating	Data suggested that men accepted programmes supporting women’s financial skills. This often improved relationships as well as increased financial planning. Involving men in the groups was noted as a positive step to foster collaboration in couples	Gender dynamics, whereby men who are unable to satisfy the culturally defined expectations to financially provide for their family find their identity challenged. This can result from displacement as well as choosing to stay in an area with high levels of violence
Falb et al. (2022), Myanmar and Democratic Republic of Congo	Interviews *n* = 51 men, *n* = 52 women; focus groups *n* = 22 men, *n* = 23 women. DRC: Men aged *M* = 43, SD = 17, education *M* = 6 yrs.; 90% married, 10% single/ widowed. Women age *M* = 35, SD = 14, education *M* = 4 yrs.; 71% married, 12% single, 10% widowed. Myanmar: men aged *M* = 32, SD = 15, education *M* = 6 yrs.; 63% married, 37% single or widowed. Women aged *M* = 45, SD = 16, education *M* = 7 yrs.,; 56% married, 44% single or widowed	In-depth interviews using and open-ended vignette approach. Focus group conversations were also held in DRC, but not in Myanmar, due to concerns about participant safety	Thematic analysis	To explore the shared risk factors and social norms underpinning the co-occurrence of violence in various forms, including IPV	Risk of domestic violence impacted by factors within and external to the home, such as power imbalances and gender expectations, exposure to armed conflict and substance use. Lack of support services and societal acceptance of violence against women exacerbate the problem	Economic and financial instability due to conflict resulted in hunger, irritability and IPV perpetration. In some cases, this was exacerbated by men needing to endure physically taxing work for little reward and taking their frustration out on their wives
Finley et al. (2010), United States	Participants (*n* = 19) were male veterans aged 23–40 (median age 28) who had served in Iraq (*n* = 14), Afghanistan (*n* = 2) or both (*n* = 3) and 11 spouses. 16 veterans had been diagnosed with PTSD	Qualitative interviews within a mixed-methods study examining post-deployment stress and PTSD	Details not reported	To explore patterns in IPV perpetration among veterans, as they are understood by veterans and their families	IPV was categorised in three ways: violence committed in anger, dissociative violence and parasomniac/hypnopompic violence	Anger and emotional dysregulation, dissociation and sleep disturbance
Fitzgerald et al. (2021), Palestine.	Women (*n* = 25) aged 18–49 living in Gaza. Marital status single (*n* = 7), married (*n* = 14), divorced (*n* = 3), widowed (*n* = 1). Education reported as primary (*n* = 2), high school (*n* = 8), university degree or higher (*n* = 10), unknown (*n* = 5). Two were employed, 23 unemployed, 17 identified as refugees	Semi-structured in-depth interviews	Inductive thematic analysis	To identify barriers to formal help-seeking for IPV survivors in Gaza	Three major factors were found to influence help-seeking for refugee women in Gaza; (1) societal expectations of traditional gender norms, (2) distrust of support services and (3) acceptance of violence as a part of everyday life	Stress of conflict, including associated hardships, including economic hardships, connected to IPV perpetration
Friedman et al. (2025), United States	Adult partners of US military personnel. Most participants were female (97%), white (77%) and non-Hispanic (88%)	Secondary analysis of semi-structured in-depth interviews	Inductive-deductive thematic analysis	To explore partners’ exposure to risk of violence perpetration, firearm suicide and associated circumstances, e.g., alcohol use, firearm access	Firearm ownership was common (62%). Partners reported exposure to situations including hazardous alcohol use and threats of IPV and suicide. Participants noted barriers to prevention and intervention	Alcohol use and access to firearms
Gerlock et al. (2014), United States	Veterans from Iraq, Afghanistan and WWII, in heterosexual relationships of min. 1 year, and partners. *N* = 441 couples. A subset of 23 couples included in qualitative analysis. Veterans were aged 27–83 (median 61) partners 24–71 (median 56)	Qualitative semi-structured interviews.	Modified grounded theory, following Strauss and Corbin’s (1990, 1994) coding procedure	To understand the long-term impacts of combat exposure for veterans and their family post-deployment.	Veterans with PTSD experience multiple interrelated issues, e.g., disabilities, substance use issues, aggression and subcultural norms. These factors add stress to relationships and can lead to IPV. Couples’ response, mutuality, locus of control balance, weakness tolerance, impact, ability to communicate and resolve conflict	Relationship dynamics, e.g., new caring responsibilities due to veterans’ deployment-related disabilities, were described as a factor that could increase veterans’ vulnerability to experiencing abuse and control. Partner’s knowledge of veterans’ capacity to harm and access to weapons created significant fear and anxiety
Guruge et al. (2017), Sri Lanka	Women (*n* = 15) survivors of IPV and service providers (*n* = 15). Survivors were aged 18–55; *n* = 8 Tamil, *n* = 5 Moor and *n* = 2 Sinhalese. Most (*n* = 11) still married to abusive partners. Remainder remarried (*n* = 2) or living with family (*n* = 2)	Qualitative interviews	Inductive thematic analysis	To explore women’s experiences of and responses to IPV in the Eastern Provence of Sri Lanka and to explore the availability of IPV services and perceptions of service providers	Respondents perceived IPV as related to war trauma. Violence used to control and enforce gender stereotypes. Service providers reported confidence about supporting survivors, but survivors perceived limited support. Survivors believed that accessing supports and leaving relationships would increase community violence and social exclusion	Post-war social changes, i.e., economic consequences of war involved a reversal of gender roles, men frustrated by perceived loss of masculinity due to unemployment; women who embraced increased autonomy perceived as violating social norms. These factors perceived as precursors to men using IPV to regain control. Alcohol problems and addiction were seen to exacerbate IPV severity
Horn et al. (2014), Sierra Leone & Liberia	Adult women receiving support services from the International Rescue Committee (IRC). Focus groups (*n* = 110), interviews (*n* = 20). Interviewees aged 18–49, *M* = 30	Focus groups and interviews	Thematic analysis	To explore women’s perceptions of the causes IPV and how they understand these causes to interact with the experiences of war	IPV linked with financial dependence on men, traditional gender expectations and war-related social change. War perceived to increase IPV in some cases due to the normalisation of violence. Sometimes war resulted in more economic activity for women, which some reported as reducing IPV due to reduced pressure on men to provide for families. Economic independence and NGO supports also increased options to leave violent relationships	Economic factors and traditional gender roles, the normalisation of violence during war
Kaul et al. (2024), Afghanistan	Adult women who had experienced domestic abuse (*n* = 60), male community members (*n* = 60), service providers (*n* = 16 men, *n* = 14 women)	Qualitative interviews	Framework thematic analysis	To explore (1) experiences of women who experienced domestic abuse and mental health problems, (2) how women, men and support workers perceive and respond to domestic violence and mental health (3) provision of mental health services for women who experience abuse	Women reported multiple and compounding traumatic experiences of violence, armed conflict and psychosocial concerns. All women reported experiencing negative mental health outcomes from abuse, which were compounded by widespread social stigma and gender norms. Support and service provision were perceived to be insufficient to meet the existing level of need	War-related mental distress and mental health problems caused by the economic impacts of war. Lack of mental health services compounds this problem
Kattoura (2022), Israel	Participants (*N* = 36) described as Arab women survivors of IPV and/or family violence, living in shelters (*n* = 27) and family violence prevention centres (*n* = 9) in Israel. Age 21–10 (*M* = 22). Married (*n* = 11), divorced/separated (*n* = 23), engaged (*n* = 1), single (*n* = 1)	Qualitative semi-structured interviews	Discourse analysis (Starks and Brown Trinidad, 2007)	To explore how the loss of land and resulting contemporary circumstances for Palestinian people in Israel has impacted men’s attitudes towards women	Experiencing partner or family violence resulted in participants experiencing fear, isolation and rumination on lack of power within society. Reported that participants described social context as (1) male-dominated and oppressive, (2) victimised and controlled by oppressors. Violations against Palestinian people reported as being projected onto women	Structural oppression and political violence being projected inward into the private sphere in intimate relationships
Kelly et al. (2012), Democratic Republic of Congo	Participants (*n* = 86) were men (*n* = 41) and women (*n* = 45). Mean age 35.6 (men), 37.5 (women). Women seeking services from a hospital-based sexual violence service. Men had attended programme accompanying a family member. Some additional participants included from the wider community	Qualitative focus groups (10), group size ranging from 6 to 11	Thematic analysis	To understand the experiences and perceptions of rape survivors and family members and explore the complex dynamics that characterise community responses to sexual violence	Consequences of rape surpass immediate reactions to the incident. Social stigma can have a long-lasting negative impact on survivors, with relatives experiencing shame and anger relating to the assault, resulting in ongoing negative experiences for survivors	IPV can be the result of a cycle of blame and anger that develops after a woman is raped in the context of conflict. Respondents reported that husbands may blame their wives and accuse them of not resisting rape. Husbands may also be blamed by wives for being unable to protect them. These dynamics may be reinforced by gender norms whereby a woman is seen as property and sex outside of marriage devalues a woman
Kiconco and Nthakomwa (2018), Uganda	Women ex-abductees (*n* = 40). All were < 18 when abducted, spent 1 yr. + in captivity and had children by LRA members. Focus groups also conducted with key informants	Structured and semi-structured qualitative interviews with both groups and individuals. In-depth interviews were carried out with 40 women	Not reported	To examine how stigma against women who return after being abducted by the Lord’s Resistance Army influences the prospect of choice in marriage upon reintegration into Ugandan society	Societal norms and cultural expectations result in ex-abductees being stigmatised and viewed as unworthy of marriage. This lesser social status reduces the social and economic opportunities when women attempt to reintegrate into society	Social stigma towards women who experienced sexual violence during abduction – viewed as less valuable and mentally unstable. Alcohol consumption exacerbates abusive behaviour. Women abductees experienced mental health consequences, including emotion regulation difficulties, which could also increase relationship aggression
Kohli et al. (2015), Democratic Republic of Congo	Participants (*N* = 18) include female survivors (*n* = 13) and male perpetrators (*n* = 5) of IPV who live in a rural village affected by prolonged conflict	Qualitative interviews	Grounded theory as adapted by Charmaz (2006)	To explore risk factors, consequences and responses to IPV in post-conflict Democratic Republic of Congo (DRC)	IPV has multiple negative impacts on survivors and children, sometimes leading to harmful coping strategies. IPV linked to husbands’ alcohol use, economic instability and gendered power dynamics	Lack of employment and financial resources is perceived to increase tension. This, as well as social gender role changes, was perceived to result in increased IPV perpetration
Lukasiak et al. (2024), Uganda	Adult women refugees (*n* = 13), residing in the refugee camp, who had experienced GBV. Mostly from DRC, with one respondent from Burundi	Individual semi-structured interviews	Content analysis (Graneheim and Lundman, 2004)	To explore women’s perceptions and experiences of GBV in a refugee camp setting in Uganda, to enhance understanding of the dynamics and risk factors for GBV in the context of displacement and refugee camps	High levels of GBV reported. Increased marginalisation and lack of resources, compounded by shifts in gender roles in camps, where women may assume provider roles, increased risk of violence. Women described extensive IPV often connected to new gendered power dynamics and control of resources	Gender role changes and shifting power dynamics in camp settings. Conflict sometimes precipitated by men striving to maintain dominance, alcohol use and appropriation of household resources
Makuch et al. (2021), Brazil	Venezuelan migrant women (=111) aged 18–49 (M 34.4, SD 11.8), living in shelters between 1–30 months	Focus group discussions with 5–14 participants	Inductive thematic analysis (Braun and Clarke, 2006)	To understand the experiences of Venezuelan migrant women living in Brazil regarding violence as part of everyday life	Physical and psychological violence is commonplace within Brazilian shelters. Violence included several forms, including xenophobia experienced when leaving the shelter	Rigid gender role expectations
Mannell et al. (2021a), Afghanistan	Participants (*n* = 20), women, aged 18–41, who had experienced sexual violence in Afghanistan and were now living in safe houses for 1 month–4 years	Qualitative semi-structured interviews	Thematic network analysis following the procedure outlined by Attride-Stirling (2001)	To explore women’s lived experience of domestic violence and conflict in Afghanistan	Domestic violence in Afghanistan perceived as resulting from both the loss of patriarchal support and the prominence of the drug trade as a result of conflict. Poverty due to conflict also increased vulnerability to violence	Social changes resulting from conflict, including poverty and the related issues, violence and addiction associated with the drug trade
Restrepo et al. (2024), Columbia	Adult ever-partnered women (*n* = 47) who had experienced armed conflict violence recruited through women’s organisations	In-depth interviews	Thematic analysis	To explore women’s experiences of multiple forms of armed conflict violence and IPV and examine pathways between these two types of violence	Armed conflict amplified patriarchal norms and intensified men’s expression of hypermasculinity through violence. Rules imposed by armed groups excused IPV if women did not comply with their traditional gender roles. Husbands blamed survivors of conflict-related sexual violence, which intensified IPV. Armed conflict generated high levels of stress that contributed to IPV	Patriarchal norms and enforcement of traditional gender roles. Control over communities by armed groups extended to control over women and justified armed conflict. Shame and stigma associated with sexual victimisation contributed to IPV. Increased levels of stress related to conflict also played a role
Wachter et al. (2018), South Sudan, Kenya and Iraq.	Participants (*n* = 284) aged 18–46. Interviews (*n* = 39) with IPV survivors, group discussions with community members (*n* = 169), community leaders (*n* = 43) and service providers (*n* = 30)	Individual interviews and focus groups	Thematic analysis (Braun and Clarke, 2006)	To explore possible risk and protective factors for IPV against women in displacement	IPV was common across camps. Risk increased in displacement due to factors such as gender norms, destabilisation of gender roles, male substance use, women’s separation from family and rapid marriages and forced marriages	Displacement-related changes in gender norms, e.g., where women’s newly acquired education was threatening to men, or women’s perceived adoption of liberal values. Men’s unemployment contributed to perceived failure to meet gender role expectations. Male substance use also reported as associated with IPV. Lack of social support due to displacement. Quick or early marriage due to economic circumstances also identified as contributing to IPV
Wirtz et al. (2014), Columbia	Adult women survivors of GBV (*n* = 35) who had been internally displaced. GBV service providers (*n* = 31) working in psychosocial support, healthcare, protection, women’s programmes and humanitarian programmes	Interviews and focus groups	Grounded theory	To understand the contexts of conflict, displacement and dynamics with GBV in Columbia	Survivors described a range of GBV (including physical, sexual, psychological and reproductive) across conflict and displacement settings, perpetrated by armed actors as well as intimate partners. Barriers to reporting and accessing supports were reported by both survivors and providers	Loss of financial stability due to displacement increased vulnerability. Some women experienced blame and violence by partners for the decision to move. Some men also blamed and abused women for experiencing sexual violence from guerrillas. Guerrillas acting as law enforcement meant no recourse for IPV
Zannettino (2012), Australia	Liberian refugee women living in South Australia (17 focus groups with 8–10 women in each). In addition, the researchers engaged in consultation with leaders of the Liberian community. Participants were aged 18–43, (*M* = 26)	Focus group discussions	Inductive thematic analysis	To understand the needs of women and children impacted by domestic and family violence from the Liberian Refugee Communities in South Australia, to inform support services	The disruption of traditional gender roles can impact domestic violence. Women’s experience of domestic violence should be contextualised with their exposure to acute and prolonged stressors of war, loss and displacement	Migration-related stressors, e.g., changing gender-role norms, can make men feel threatened by women’s increased autonomy. Poverty and economic stress can contribute to men’s fear of losing status, which was perceived as precipitating IPV. Stress compounded by experiences, e.g., living in a conflict area, experiencing violence, needing to flee, grief of losing family members, as well as living in transient accommodation

**Table 2.** Summary of quantitative studies included in the review Table 2. long description.

Authors, year, country	Sample details	Reported IPV	Study design	Analysis	Study aims/objectives	Main findings	Proposed driver
Botchkovar et al. (2025), Ukraine	Random sample (*n* = 1,185) Ukrainian adults, women (*n* = 655) and men (*n* = 530), aged 18–94, *M* = 46, SD = 17; 53% married, 50% employed	Measured with Likert response items capturing frequency of carrying out abusive acts: verbal abuse, pushing/ shaking partner, slapping or twisting arm, or using force	Cross-sectional survey	Negative binomial regression models with interaction terms to assess moderation effects	To examine the impact of exposure to the Donbas war on IPV among men and women, relative to alcohol use, daily stressors and negative emotions	Daily stressors, negative emotions and alcohol use predicted IPV for both genders. War exposure did not have a direct relationship with IPV. However, both direct and indirect exposure conditioned effects of negative emotions, alcohol abuse and daily stressors on IPV	Daily stressors, negative emotions and alcohol use
Bourey et al. (2024), Democratic Republic of Congo	Baseline data from a GBV survey, including all men (*n* = 2080) who provided data on variables of interest for analysis	Items capturing behaviours in the most recent relationship (e.g., hitting, slapping, kicking, forced sex). Those endorsing items were asked to report on previous 12 months to define group of past year perpetrators	Cross-sectional survey	Structural equation modelling	To examine how potential risk factors (interpersonal violence exposures, trauma symptoms and gender inequitable attitudes and socioeconomic adversity) for IPV relate to each other and to use of IPV	Interpersonal violence associated with IPV and gender inequitable attitudes. Gender attitudes were also linked to the use of IPV. Interpersonal violence was linked to trauma symptoms, which were linked to IPV. Use of IPV decreased as conflict exposures increased, and there was no path from socioeconomic adversity to IPV	Interpersonal violence exposures, trauma symptoms and gender inequitable attitudes
Cesur and Sabia (2016), United States	Dataset 1 (Add Health) = 476 active duty male soldiers, aged 24–34. Dataset 2 (OD HRB) = 11,474 active duty service men aged 18–50	Dataset 1: yes/no items for threatening, hitting and injuring endorsed by 5.7%; 3.2% and 1.7% respectively. Dataset 2 used single item, endorsed by 1.7%	Quantitative econometric analysis of two secondary datasets	Linear regression	To examine whether conflict exposure in military deployment is associated with domestic violence perpetration	Soldiers exposed to combat were more likely to perpetrate domestic violence than those deployed to non-conflict zones. The strength of the relationship reduced when controlling for mental health and substance use problems	Mental distress and substance use problems induced by direct exposure to combat
Creech et al. (2017), United States	Women US Army veterans deployed to Iraq/Afghanistan (*n* = 102). Mean age 36, SD = 9). Mean yrs. education 15.99 (SD = 2.99). 77% White, 10% Black, 2% Multiracial, 2% Native American, 6% Other. 60% married, 1% separated, 3% divorced, 19% dating, 9% engaged, 9% single. 88% employed	63% reported victimisation, 73% reported perpetration in the past 6 months, assessed using the revised CTS (CTS2; Straus et al., 1996)	Cross-sectional quantitative	Multiple linear regression analysis	To examine factors associated with IPV use and experience among US women veterans deployed to Iraq and Afghanistan	Relationship satisfaction associated with psychological IPV experience; alcohol issues and psychological IPV experience associated with physical IPV use and experience and sexual IPV experience. Sexual harassment in deployment associated with psychological IPV experience	Mental health problems and impairments to relationship functioning caused by exposure to conflict-related trauma
Chiu et al. (2022), United States	Veterans (*n* = 217) deployed to Iraq, and cohabiting partners. Age *M* = 36 (SD = 5.94), 95% male. Race 78% White, 11% Black, 1% Asian, 0.5% Native American, 0.5% Hawaiian, 5% Other, 13% Hispanic. Education: 7% high school, 49% some college, 15% college graduate, 7% advanced degree	IPV over the past 6 months was assessed using the 78-item Conflict Tactics Scale–2 (CTS2; Straus et al., 1996), which was administered to warzone veterans and their partners – took greater of the two scores	Longitudinal quantitative	Regression	To examine the associations between post-deployment neurocognitive performance and subsequent IPV (5–13 years later)	Greater psychopathology and history of TBI were associated with more frequent psychological IPV perpetration. Poorer post-deployment neurocognitive performance predicted higher subsequent psychological and physical IPV perpetration, adjusting for demographics, psychological health and TBI	Psychopathology (PTSD, depression, problematic alcohol use), TBI, neurocognitive problems
Gerlock et al. (2016), United States	Male army veterans in treatment for PTSD (all met DSM-IV TR criteria), in an intimate heterosexual relationship for at least a year. Also surveyed their partners. Total sample *n* = 882	Self and partner reports, using Relationship Behaviour Inventory and Abusive Behaviour Inventory (ABI questionnaire). 43% of men had perpetrated IPV	Cross-sectional quantitative	Discriminant function analysis	To examine factors that might differentiate veterans with PTSD who are violent to their partners from those who are not	Only the level of relationship mutuality significantly differentiated IPV perpetrators from non-perpetrators no effect found for childhood witnessing of IPV, substance use or demographic variables	Relationship mutuality and PTSD symptom severity. The ability of a couple to talk about and process conflict-related experiences together may encourage better outcomes
Gibbs et al. (2021), Palestine	Adult women living in the Occupied Palestinian Territories (OPT; *n* = 534). 68% (*n* = 365) were currently married, and 23.% had never been married. 40% were aged 18–29, 44% aged 30–49 and 17% aged 50+. 30% completed secondary education and a further 39% had some secondary education	Behaviourally specific emotional, physical and sexual violence items, drawing on the WHO Multi-Country Study on Domestic Violence methodology. Rates of violence reported as 28% emotional abuse, 25% physical and 24% sexual	Cross-sectional quantitative	Latent class analysis and structural equation modelling	To describe patterns of occupation-related events among women living in OPT, and to assess factors associated with these patterns, health impacts and pathways through which occupation-related events are associated with IPV	Three classes of exposure to occupation-related events (ORE): (1) multiple events directed at women, their families and homes, (2) violence against family members and homes and (3) relatively few direct experiences of ORE. Group membership associated with IPV experience and depression. ORE increased IPV experience via gender inequitable attitudes, depressive symptoms and relationship conflict	Occupation-related events, gender inequitable attitudes, depression and relationship conflict.
Gupta et al. (2012), South Africa	Men (*n* = 772), aged 18–29 (12%), 30–39 (24%), 40–49 (25%), 50–59 (25%), 60 + (15%). Race reported as Black (77%), Coloured (17%), White (1.5%), Indian (4.5%). Education: none (12%), Grades 1–11 (61%), Grade 12 (13%), Grades 13+ (15%). 51% employed, 49% unemployed. 67% married, 25% partnered, 8% separated, divorced or widowed.	Men reported physical IPV perpetration, using behavioural items from WHO Mental Health Survey. All responses regarding physical IPV perpetration were dichotomised as never/ever.	Cross-sectional quantitative	Logistic regression	To examine associations between human rights violations and IPV in South Africa – analysed those who supported liberation and those who supported the government separately	In liberation supporters, a significant association between experiencing major HRV, custody-related HRV, victimisation of close friends/family and physical IPV perpetration. Among government supporters, significant association between experiencing HRV and victimisation of close friends/immediate family and IPV perpetration	Experiencing human rights violations
Heavey et al. (2017), United States	US Army Reserve and National Guard soldiers and partners, 257 couples in opposite-sex relationships, *n* = 246 male soldiers (mean age 33.3 SD = 6.2) and *n* = 246 their female partners (mean age 32 SD = 6.5); *n* = 33 female soldiers (mean age 34.3 SD = 5.9) and their male partners (mean age 33.2 SD = 4.7)	Conflict Tactics Scale (Straus et al., 1996). Used the highest of self and partner reports to estimate max rate. Violence perpetration: Sexual 15% (men), 12% (women). Physical 18% (men) 24% (women). Injury 6% (men), 9% (women)	Cross-sectional quantitative (drawn from a longitudinal study, but the article presents data from the first wave only)	Logistic regression	To examine the impact of combat exposure on IPV	Combat exposure did not increase the likelihood of relationship aggression, but it was associated with IPV resulting in injury where it did occur.	Direct combat exposure associated with more injurious violence, possibly due to military socialisation.
Iverson et al. (2020), United States	US women veterans deployed to Iraq/Afghanistan undergoing TBI evaluation (*n* = 127); Age *M* = 37.4; Race/ethnicity 58% White, 18% Hispanic; Relationship status 59% married/partnered, 18% single, 23% separated/widowed/divorced; Sexual orientation 81% heterosexual, 19% other/undisclosed.	Humiliation, Afraid, Rape, Kick (HARK) screener (Sohal et al., 2007 four yes/no items assessing psychological violence, fear of partner/ex, sexual violence and physical violence. Lifetime IPV reported by 63%.	Cross-sectional quantitative	Chi-square and t-tests	To examine the potential association between traumatic brain injury (TBI) and IPV exposure in female US Army veterans.	No significant association between clinician-confirmed TBI, but significant associations found between lifetime IPV and neurobehavioural symptoms, substance use and back pain.	Neurobehavioural symptoms, substance use and back pain may act as vulnerability factors in conflict-exposed army personnel, but directionality of relationship unclear.
Jewkes et al. (2017), Papua New Guinea	Random sample of adults, *n* = 746 men, *n* = 793 women, Bougainville, Papua New Guinea, age 18–49. Women *M* = 31.4, men = 31.5. Most had some schooling, majority primary only. 48.1% of women, 73.7% men employed. Two thirds married/cohabiting; 26.9% men, 16.7% women never partnered/ currently unpartnered.	Items based on WHO Multi-Country Study survey. 33.3% of women had experienced physical or sexual partner violence in the past year, and 34.3% of men reported perpetrating IPV	Cross-sectional quantitative	Multiple regression	Describe conflict experiences, explore perceptions of enduring impact of conflict and examine associations between these impacts and mental ill-health and violence against women.	War trauma, mental health and substance problems were prevalent. War trauma related to PTSD (women) and PTSD, alcohol problems and depression (men). Impact of conflict related to depression (both genders); problem drinking, suicidal thoughts (women); drug use (men). Impact of conflict related to rape and physical and/or sexual partner violence.	Perceived enduring impact of conflict and mental health effects in men, e.g., problem drinking, depressive symptoms drug use, affected likelihood to perpetrate. The higher the perceived enduring impact, the larger the effect on IPV.
Kar and O’Leary (2013), United States	Male veterans (*n* = 110) deployed to Iraq or Afghanistan. Age *M* = 36.98 (SD = 9.93). All married or partnered. Education 38% high school, 56% some college, 6% some graduate training. Ethnicity, 68% White, 32% identified as minorities, e.g., Hispanic/Latino (17%), African American (6%).	Physical assault subscale of CTS2	Cross-sectional quantitative	Path analysis	Examine relationships between PTSD, IPV, emotional intimacy and relationship satisfaction.	A third of veterans had clinically elevated PTSD scores, and 31% reported perpetrating past-year physical IPV. Poor emotional intimacy mediated the association between PTSD symptoms and IPV. Past IPV perpetration was associated with current IPV perpetration.	Poor emotional intimacy.
Lane et al. (2022), United Kingdom	Military personnel who had deployed to Iraq and/or Afghanistan (*n* = 4,566), drawn from a large cohort study on health and well-being of military personnel. Male *n* = 4,146, female *n* = 420, mostly partnered (*n* = 3,978), majority educated to A-levels (*n* = 3,196).	Two questions used to capture two IPV outcomes: arguing and being physically violent towards partner.	Cross-sectional quantitative	Univariate and multivariate logistic regression analyses	To estimate the prevalence of and risk factors for post-deployment relationship conflict and partner violence.	Relationship conflict reported by 34.7% and physical IPV reported by 3.4%. Men more likely to report relationship conflict, while reported IPV perpetration was similar among men and women. For men, risk factors for relationship conflict and IPV included being in the army, childhood adversity, military trauma and mental health and alcohol problems.	Childhood adversity, military trauma, mental health and alcohol problems
Orcutt et al. (2003), United States	Male US Army Veterans from US/Vietnam war and female partners. Data from wider study (US National Vietnam Veterans Readjustment Study), *n* = 376 couples. Racial/ethnic identity of veterans was 24% African American, 29% Latino, 47% White/Other, with a similar distribution for partners.	Past-year IPV assessed by CTS (Straus, 1979).	Cross-sectional quantitative	Structural equation modelling	To examine the impact of early-life stressors, war-zone stressors and PTSD symptom severity on partner reports of recent male-perpetrated IPV.	IPV associated with early adversity, warzone stressors and PTSD. Early family adversity, childhood antisocial behaviour and war-zone stressors indirectly associated with IPV via PTSD. The warzone stressor of perceived threat had direct and indirect (through PTSD) relationships with IPV.	PTSD symptoms
Rees et al. (2018), Timor-Leste	Women and male partners (*n* = 870). Women’s age *M* = 29, 17% no school or primary only, 58% completed junior or senior high school, 25% had a degree or other qualification, 52% unemployed. Men’s age *M* = 34, 51% finished junior or senior high school, 31% had obtained further qualifications. 34% employed.	WHO Violence Against Women Interview; 12-month rate of IPV, excluding sexual IPV. 60.8% (*n* = 529) of women fell into the severe abuse category; the remainder were grouped into a low or no abuse category.	Cross-sectional quantitative	Structural equation modelling	To examine whether exposure to torture plays a role in causing IPV perpetration, and whether this relationship is mediated by torture-related trauma.	Men’s experience of torture directly associated with IPV perpetration, and also indirectly via mental disturbance (i.e., hazardous drinking and PTSD). Lower socio-economic status and younger age are also associated with torture exposure.	Mental disturbance (i.e., PTSD, severe psychological distress and hazardous drinking).
Rojczyk et al. (2024), United States	Male US Army veterans (*n* = 49) who had deployed to Iraq or Afghanistan, aged 21–55 (*M* = 33, SD = 8). Majority white (*n* = 41; 84%).	Psychological and physical assault subscales from CTS-2.	Neuroimaging (diffusion-weighted magnetic resonance imaging; dMRI)	Descriptive statistics, odds ratios and partial correlations	To investigate whether IPV perpetration is associated with limbic abnormalities, and to test associations with PTSD, depression, substance use disorder, mild traumatic brain injury (mTBI) and war-related stress.	Veterans with PTSD, depression, substance use disorder or mTBI had higher odds of perpetration. Greater war zone-related stress, PTSD symptoms, depression and mTBI were significantly associated with perpetration. Psychological aggression was associated with higher fractional anisotropy tissue in the right amygdala-hippocampus complex.	PTSD, depression, substance use problems, mild traumatic brain injury (mTBI)
Saile et al. (2013), Uganda	Data from a survey of children and guardians from war-affected communities. Subsample of 235 guardian couples. Ages 20–88, women *M* = 36 (SD = 9), men *M* = 42 (SD = 11). 32% women and 3% of men had no education, 64% women and 68% men attended some primary school, 3% women and 16% men had completed primary school, 4% women and 9% men had some secondary school, 4% men and no women had some further education.	Composite Abuse Scale (CAS; Hegarty et al., 2005), 86% reported at least one type of past-year abuse. 43% afraid of partners, 80% experienced psychological abuse, 71% physical abuse, 52% experienced isolation and 23% experienced sexual violence. 72% ≥1 incident of physical or sexual abuse.	Cross-sectional quantitative	Descriptive statistics, correlations, regression	To assess the prevalence and risk factors of partner violence experienced by women in conflict-affected communities in Northern Uganda.	Women’s prior exposure to war-related traumatic events, women’s re-experiencing symptoms and men’s level of alcohol-related problems were associated with higher levels of partner violence against women. Differential effects of the predictor variables emerged with respect to different subtypes of partner violence.	Women’s war-related trauma and re-experiencing symptoms, and men’s alcohol-related problems
Sileo et al. (2021), Liberia	*N* = 183 pregnant women accessing community healthcare in Monrovia, Liberia. Data from a larger study on sexual and mental health in pregnant women. Ages 18–30 (*M* = 22.78, SD = 3.68), between 13–24 weeks of gestation.	Severity of Violence Against Women Scale (Marshall, 1992) used to measure past-year violence. Lifetime experience of IPV endorsed by 45%.	Cross-sectional quantitative	Mediation – path analysis	To examine potential mediators between trauma (including war-related trauma) and IPV experience in pregnant women in post-conflict Liberia	More trauma exposure was associated with more severe experience of IPV. Relationship between trauma and IPV was significantly mediated by anxious attachment and attitudes justifying violence.	Anxious attachment style and attitudes towards norms relating to IPV
Snir et al. (2017), Israel	Israeli male army veterans (*n* = 230) – data gathered in 2004. Approx. 50% held in captivity for some months. Age *M* = 53.6 (SD 4.56); education *M* = 14 yrs. (SD 3.45); 93.4% married; 61.7% self-described as secular.	Index of verbal and physical aggression using Conflict Tactics Scale – descriptive not reported.	Cross-sectional quantitative	Hierarchical regression analysis	To examine predictors of ‘aggression directed inward’ i.e., suicidality, or ‘directed outwards’, i.e., partner violence.	Aggressive impulses drive both suicidality and IPV. Suicidal ideation related to aggressive impulses in the presence of PTSD or under high guilt; paranoid ideation buffered these effects. Partner violence related to aggressive impulses under low guilt and absence of PTSD.	Aggressive impulses that arise during wartime. Only found to be significant in the presence of low guilt and absence of PTSD.
Taft et al. (2005), United States	Subsample of *n* = 109 men from a wider study of army veterans. Ethnicity *n* = 70 (64%) Caucasian, *n* = 28 (26%) African American, *n* = 9 (8%) Native American, *n* = 1 (1%) Asian, *n* = 1 (1%) other; *n* = 35 (32%) identified as Latino–Hispanic. Age *M* = 40, SD = 3. Female partners reported on IPV – demographics similar to males. 89% married.	Past-year physical abuse, measured with partner ratings on the eight-item Violence subscale of the CTS (Straus, 1979).	Cross-sectional quantitative	Classification tree analysis – optimal discriminant analysis	To examine group differences in potential risk factors between partner-violent veterans with or without PTSD, and non-violent veterans with PTSD.	Partner-violent male veterans with PTSD were higher than other groups on all risk factors considered, apart from family of origin violence.	Traumatic warzone experiences, comorbid psychiatric problems (alcohol use problems, depression, combined with PTSD) and relationship factors (marital adjustment).
Taft et al. (2007), United States	US veterans (*n* = 60), in relationships for ≥1 yr.; *n* = 18 met criteria for PTSD; *n* = 42 PTSD-negative. PTSD-positive group aged *M* = 51 (SD = 4); PTSD-negative group aged *M* = 52 (SD = 5). Most PTSD-positive (83.3%) and PTSD-negative (88.1%) participants were white. All PTSD-positive participants and 95.2% of PTSD-negative participants had at least a high school education.	The CTS (Straus, 1979) used to measure physical and psychological aggression perpetration. Approx. 40% of participants reported at least one act of physical aggression, 91% reported psychological aggression.	Cross-sectional quantitative	ANOVA and mediation analysis.	To examine relationships between PTSD symptoms, anger and partner violence perpetration.	Participants with PTSD had higher state anger over time. In a trauma-prime condition, participants with PTSD demonstrated increased anger reactivity compared with PTSD-negative participants. PTSD symptoms associated with physical assault and psychological aggression perpetration and trait anger mediated these relationships.	Anger
Taft et al. (2015), United States	Male US Army veterans (*n* = 92) who had been deployed to Iraq or Afghanistan and who had been exposed to combat. All partnered within the previous 6 months, no current psychosis or addiction. Age *M* = 40, SD = 10.	Physical assault subscale of the CTS-2, 28% of the sample reported past-year perpetration.	Cross-sectional quantitative	Correlation and mediation analysis	To examine social information processing factors that could represent pathways through which PTSD symptoms relate to anger and IPV perpetration.	Hyperarousal was the PTSD symptom cluster most strongly associated with anger and IPV. Mediation hypothesis partially supported. Hostile attribution biases associated with IPV perpetration.	PTSD, cognitive biases and hostile attribution biases
Teten et al. (2010), United States	Male army veterans (*n* = 86) deployed to Vietnam or Iraq/Afghanistan, screened for PTSD in a clinic. All in heterosexual relationships for 3+ months; 41% White, 34% African American, 24% Hispanic, 1% Asian American; 57% married; 19% on active duty, 34% spent ≥1 year in combat.	The CTS-2 (Straus et al., 1996) used to record past-year psychological, physical and injurious IPV. Reported 40% physical aggression.	Cross-sectional quantitative	Chi-square, correlations, Fisher’s exact test, t-tests, odds ratios and confidence intervals.	To examine the potential association of PTSD and IPV, as well as potential differences between groups deployed in different military operations.	Veterans deployed to Iraq or Afghanistan with PTSD were significantly more likely to report psychologically abusing their partner than veterans without PTSD. Veterans with PTSD were also more likely to sustain injury from their partners than those without PTSD.	PTSD
Tharp et al. (2016), United States	Male veterans (*n* = 100) and female partners seeking couples therapy. 82% veterans and 79% partners White, 9% Black, 2% Hispanic, 4% Indian and 3% and 6% other races/ethnicities. Veterans’ ages 21–51 (*M* = 31.3, SD = 7.4). Partners 19–56 (*M* = 30.0, SD = 8.1). 85% married, 15% cohabiting. Average education 14.0 (SD = 1.3) yrs. for veterans;14.7 (SD = 1.7) yrs. for partners. 64% and 67% of veterans and partners employed.	The therapy programme screens out those with an active substance use problem or who screen positive for severe IPV. Study measured past-year IPV as measured by a combination of items from CTS and the CTS-2	Cross-sectional quantitative	Descriptive statistics, MANOVA and kappas	To quantify and describe IPV in relationships of US army veterans seeking couple’s therapy, to compare rates across genders, to examine correspondence of reports within relationship dyads, and to examine role of male veteran PTSD	Most couples reported verbal aggression. Men perpetrated more sexual coercion, women more physical aggression. Correspondence in reports varied by type of violence from high (verbal aggression) to low (sexual coercion). Three patterns identified: verbally aggressive, one-sided physical aggression and mutual physical aggression. Mutually aggressive couples reported most frequent violence. IPV frequency and pattern unrelated to PTSD.	Authors proposed that veteran PTSD may have an impact but found no significant association.

As this project was funded as part of a funded research collaboration between Ireland and Uganda to develop new research collaboration networks, the findings of the review were presented at a meeting of stakeholders and activists engaged in work relating to refugee and displacement issues, and gender-based violence prevention and support, in Kampala, Uganda, in October 2024. Preliminary findings were discussed at the stakeholder meeting, with a focus on how the findings may apply to the Ugandan context. This process also served to refine and develop the findings through discussion and validation. The findings were grouped and analysed by population, separately considering military samples, refugee and internally displaced samples, and separately considering perpetration and victimisation. Possible drivers at the individual, relational and structural levels are explored.

## Results

### Study characteristics

The final search returned 49 articles. Quantitative and qualitative studies are presented separately (see Tables 1 and 2). In total, 25 included studies were qualitative and 24 were quantitative. Of the quantitative studies, all but one (Chiu et al., 2022) were cross-sectional, and the majority (*k* = 14) were conducted in the United States (US) on army veterans returning to civilian life, with relatively few studies focused on low- and middle-income countries (LMICs). Study context and sampling procedures differed substantially, including military samples from high-income countries such as the US, as well as community-based studies and household surveys in LMICs.

### Quality assessment

The quality of included studies was high overall (see Supplementary Tables 3 and 4 in Supplementary Files 2 and 3 for full details of quality assessment). Among the qualitative studies, sampling and analytic procedures were generally well-outlined and appropriate to the research questions. In a minority of cases, the qualitative methodologies and approaches to sampling were not adequately described to clearly ascertain their appropriateness to the research question. However, in most cases, the findings were well-supported by data and methods were clearly outlined and justified. Similarly, the overall quality of the included quantitative studies was high, with several articles drawing on random samples and meeting all MMAT quality assessment criteria (Jewkes et al., 2017; Rees et al., 2018; Gibbs et al., 2021; Lane et al., 2022; Botchkovar et al., 2025). Studies differed in how they conceptualised and measured conflict exposure, with some using military deployment to conflict zones as a proxy for conflict exposure, and others directly measuring exposure using dichotomous items capturing conflict-related events or standardised scales.

Across quantitative studies, methods of measuring IPV varied significantly, which presents a challenge for the comparison of findings. For example, some studies used dichotomous (yes/no) items (e.g., Gupta et al., 2012; Cesur and Sabia, 2016), while others used standardised scales, such as the Conflict Tactics Scale-2 (CTS2; Straus et al., 1996) which ask participants to report on the frequency of a range of abusive behaviours or experiences from a given time period (e.g., 6 or 12 months). A total of 10 included studies used a version of the CTS or its subscales (Orcutt et al., 2003; Taft et al., 2005; Taft et al., 2007; Kar and O’Leary, 2013; Tharp et al., 2016; Creech et al., 2017; Heavey et al., 2017; Snir et al., 2017; Chiu et al., 2022; Rojczyk et al., 2024). All of the studies using the CTS were based on samples from the US or Israel. Other measures used included the Severity of Violence Against Women Scale (Marshall, 1992; used with a sample of Liberian women by Sileo et al., 2021); the Composite Abuse Scale (Hegarty et al., 2005; used with a sample from Uganda by Saile et al., 2013). Other studies used and adapted items from large-scale surveys such as the WHO Multi-Country Study on Domestic Violence (Garcia-Moreno et al., 2006; used by Gibbs et al., 2021, in Palestine and by Jewkes et al., 2017 in Papua New Guinea).

Studies differed in relation to whether they studied one or both partners, and whether they considered victimisation, perpetration or both. Studies also differed in their operational definitions of IPV and the types of violence included for consideration. For example, some studies (e.g., Snir et al., 2017; Rees et al., 2018) omitted consideration of sexual IPV. Finally, quantitative studies also considered different time periods when asking participants about abuse. For example, some asked participants to report on the previous 6 months (e.g., Creech et al., 2017; Chiu et al., 2022), some considered past-year IPV (e.g., Taft et al., 2005; Teten et al., 2010; Saile et al., 2013; Cesur and Sabia, 2016; Jewkes et al., 2017) and others considered lifetime rates (e.g., Iverson et al., 2020).

### Armed conflict and IPV: Drivers of the association

The next sections describe findings on the factors identified by the literature as drivers of the association between armed conflict and IPV. These factors are divided broadly by population, according to the following groupings: (1) military samples, (2) populations in conflict-affected contexts and (3) refugees and displaced persons. A brief summary of drivers separated by group is provided in Table 3.

**Table 3.** Summary of drivers separated by population Table 3. long description.

Driver type	Military samples	Conflict-affected contexts	Refugee and IDP communities
Trauma, mental health and emotion regulation problems	PTSD, depression, anger, TBI, neurocognitive problems	Trauma symptoms, psychological distress	War-related mental health problems, migration-related stress
Substance use	Problematic alcohol and substance use	Problematic alcohol and substance use, drug trade issues	Problematic alcohol and substance use
Relationship	Relationship satisfaction, emotional intimacy, mutuality and changing dynamics associated with acquired disability	–	Forced or early marriage
Economic factors	–	Poverty, financial stress, livelihood loss	Camp deprivation, lack of economic opportunity
Gender dynamics	–	Gender role shifts, men’s role of ‘provider’ threatened, stigma of conflict-related sexual violence	Men threatened by women’s autonomy, adoption of new values
Social and structural factors	Access to weapons	Policing issues, paramilitarism, loss of social support and access to weapons	Discrimination experiences, language barriers, loss of social support, overcrowding and poor living conditions in camps

## Military samples

Studies based on military samples or the partners of military personnel were based primarily in the US (Orcutt et al., 2003; Taft et al., 2005; Taft et al., 2007; Finley et al., 2010; Teten et al., 2010; Kar and O’Leary, 2013; Gerlock et al., 2014; Taft et al., 2015; Cesur and Sabia, 2016; Gerlock et al., 2016; Tharp et al., 2016; Creech et al., 2017; Heavey et al., 2017; Iverson et al., 2020; Chiu et al., 2022; Rojczyk et al., 2024; Friedman et al., 2025), with one each from the UK (Lane et al., 2022) and Israel (Snir et al., 2017). These studies tended to examine individual-level variables that increased the risk of IPV perpetration and victimisation in conflict-exposed military personnel. The most commonly examined driver of the association between armed conflict and IPV perpetration across these studies was PTSD, which was examined by ten of the quantitative studies on military samples. The analysed evidence widely supports PTSD as a significant risk factor for IPV perpetration among conflict-exposed military samples from high-income countries. For example, Orcutt et al. (2003) found that PTSD symptom severity significantly mediated the relationship between warzone stressors and IPV. Similarly, Teten et al. (2010) found that US Army veterans with PTSD reported a higher level of perpetration compared with veterans without PTSD.

However, there were a few studies that potentially complicate the straightforward conceptualisation of PTSD as a risk factor for IPV. For example, in a study with Israeli military veterans, Snir et al. (2017) found that aggressive impulses were associated with both suicidality and IPV. The authors found that aggressive impulses arising from conflict exposure increased the likelihood of both suicidality and IPV; however, these associations were moderated by PTSD and guilt. Specifically, aggressive impulses were associated with suicidality in the presence of PTSD symptoms and high levels of guilt, whereas aggressive impulses were associated with IPV in the absence of PTSD and under conditions of low guilt. The authors proposed that guilt may represent the internalisation of a moral code, shaping whether aggressive impulses are turned inward on the self or outward towards others. Drawing on Lifton (1973), they further suggest that wartime produces detachment and depersonalisation in those who participate in it, reducing guilt associated with violent acts, potentially facilitating the persistence of outward aggression in the private sphere post-conflict. Tharp et al. (2016) also examined patterns of relationship aggression in US Army veterans seeking couple’s therapy and found no difference in mean aggression between those with and without a PTSD diagnosis. However, Tharp et al. (2016) conceptualised PTSD as a categorical variable, while many of the other studies examining the role of PTSD treated it as a dimensional variable, which may have impacted the results.

Problematic alcohol use was also commonly investigated as a potential contributor to the relationship between conflict exposure and IPV, reported as a significant perpetration-related factor in 12 studies across the review, and five military samples. This evidence supports the significance of alcohol as a driver of the association between armed conflict and IPV. For example, in a longitudinal study of US Army veterans, Chiu et al. (2022) found that problematic alcohol use was associated with partner-corroborated reports of both physical and psychological abuse. Other risk factors investigated in the military samples included mental health problems such as depression, attachment styles, anger and traumatic brain injury (TBI).

Some included studies directly investigated associations between exposure to armed conflict and relationship characteristics. For example, Gerlock et al. (2014) qualitatively interviewed a sample of US Army veterans and their families, identifying various relationship dynamics that were perceived by respondents as linked to aggression in their relationships. These involved situations, for example, where veterans had acquired a disability during their deployment, placing their partner in a caregiver role, thereby increasing veterans’ vulnerability to abuse. A further study by Gerlock et al. (2016) involved an exploratory analysis of a range of factors (e.g., family of origin violence, PTSD severity and demographic factors) to assess whether they differentiate veterans who were violent to their partners from those who were not. Gerlock et al. (2016) identified only the factor of relationship mutuality to significantly differentiate these two groups, a construct defined as ‘the bi-directional movement of feelings, thoughts and activity between persons in a relationship’ (*p.* 673, Gerlock et al., 2016). Similarly, a study of women army veterans from the US (Creech et al., 2017) reported that relationship satisfaction explained a significant amount of the variance in the women’s recent psychological IPV.

## Conflict-affected contexts

Studies from conflict-affected contexts included consideration of a range of geographic locations, including Cote D’Ivoire (Falb et al., 2014; Cardoso et al., 2016); Northern Ireland (Doyle and McWilliams, 2020); Myanmar (Falb et al., 2022), the Democratic Republic of Congo (DRC; Bourey et al., 2024; Falb et al., 2022; Kelly et al., 2012; Kohli et al., 2015); Sri Lanka (Guruge et al., 2017); Sierra Leone (Horn et al., 2014); Liberia (Horn et al., 2014; Sileo et al., 2021); Afghanistan (Mannell et al., 2021a; Kaul et al., 2024); Palestine (Fitzgerald et al., 2021; Gibbs et al., 2021); Israel (Kattoura, 2022); Uganda (Saile et al., 2013; Kiconco and Nthakomwa, 2018), Columbia (Restrepo et al., 2024), Ukraine (Botchkovar et al., 2025), Papua New Guinea (Jewkes et al., 2017) and South Africa (Gupta et al., 2012). Similar to the studies with military samples, several of these studies also examined the ways in which the trauma of war and armed conflict can increase the risk of IPV. For example, studies by Gupta et al. (2012), Rees et al. (2018) and Gibbs et al. (2021) found that experiencing human rights violations such as torture and occupation-related violence and loss were associated with increased risk of perpetration. These studies also investigated other mental health problems and the role of alcohol use, indicating a role of these factors in increasing the risk of IPV. For example, Rees et al. (2018) found that mental disturbance (defined as the presence of hazardous drinking, severe psychological distress or PTSD) mediated the relationship between torture exposure and IPV perpetration. Similarly to findings from the military samples, alcohol use was perceived by respondents in studies from several conflict-affected contexts to be a significant factor influencing the severity and frequency of perpetration (Kohli et al., 2015; Guruge et al., 2017; Kiconco and Nthakomwa, 2018).

There is some evidence from the studies included in this review that conflict-related trauma and PTSD can also increase vulnerability to victimisation, as well as increasing the likelihood of perpetration. For example, Saile et al. (2013) conducted a cross-sectional quantitative study examining factors affecting IPV risk in conflict-affected communities of Northern Uganda. Saile et al. (2013) found that women’s exposure to war trauma and related re-experiencing symptoms were associated with IPV experience. Somewhat similarly, Sileo et al. (2021) conducted a quantitative study of pregnant women in post-conflict Liberia and found that women’s trauma (including conflict-related trauma) was associated with the severity of their IPV experiences.

Several included studies from conflict-affected contexts highlighted how the structural and societal consequences of war and armed conflict can affect dynamics relating to IPV perpetration and victimisation. One widely investigated factor, explored in several included studies (e.g., Cardoso et al., 2016; Wachter et al., 2018; Falb et al., 2022), concerned the economic effects of conflict. For example, in a study from Côte d’Ivoire (Cardoso et al., 2016), respondents described how the conflict had affected people’s ability to earn livelihoods, resulting in problems such as poverty, lack of social support and financial stress, which were all factors perceived by respondents to affect men’s likelihood of perpetrating IPV. Similarly, Falb et al. (2022) found that economic and financial instability in the aftermath of conflict was perceived to be a major source of ongoing stress and emotional problems, which increased the risk of IPV perpetration.

Another social factor highlighted in several studies (e.g., Zannettino, 2012; Falb et al., 2014; Kohli et al., 2015; Cardoso et al., 2016; Guruge et al., 2017; Wachter et al., 2018; Daoud, 2021; Al-Natour et al., 2022) was gender relations. This was reflected, for example, in the findings of Guruge et al. (2017) from Sri Lanka, whereby social changes associated with the conflict were reported as resulting in men’s traditional social roles being threatened, contributing to resentment towards their partners. Falb et al. (2014) also found that men’s frustration at not being able to fulfil their perceived gender role of providing financially for their families resulted in feelings of shame, which in turn increased the risk of IPV.

Some studies highlighted the impact of gendered social stigma towards women who survived conflict-related sexual violence (e.g., Kelly et al., 2012; Kiconco and Nthakomwa, 2018; Daoud, 2021). For example, a qualitative study from the Democratic Republic of Congo (DRC) by Kelly et al. (2012) reported a dynamic whereby women who survived conflict-related rape experienced blame from their husbands as well as social ostracisation; respondents described how survivors were sometimes accused of failing to resist the rape, and how rape can result in women being socially devalued due to gendered social norms. A study from Uganda (Kiconco and Nthakomwa, 2018), based on interviews with women who were abducted by the Lord’s Resistance Army (LRA), identified a similar pattern, where women who experienced sexual violence experienced stigma, shame and ostracisation on their reintegration into society.

The intersection between gender role changes and economic factors was noted in multiple studies from conflict-affected and displacement contexts (e.g., Kohli et al., 2015; Cardoso et al., 2016; Daoud, 2021; Al-Natour et al., 2022). Respondents from focus groups with conflict-affected communities in Côte d’Ivoire (Cardoso et al., 2016) described how the conflict created a necessity for some women to be more economically independent. Respondents reported that this change in gender roles, coupled with men’s difficulties in securing employment post-conflict, was a factor that increased IPV. Daoud (2021), in a study of Syrian refugee women in Jordan, reported that men’s failure to economically provide for their families was a source of stress and shame, which resulted in increased IPV perpetration. Similar dynamics were reported by Kohli et al. (2015) in the Democratic Republic of Congo and Al-Natour et al. (2022) in Jordan. Mannell et al. (2021a), in their study of women seeking refuge from domestic violence in Afghanistan, also found that the ways in which the conflict affected the social fabric and norms of daily life were central to the women’s understanding of how the conflict influenced their experience of IPV. Respondents interviewed by Mannell et al. (2021a) described how the loss of trusted patriarchal support, issues relating to addiction and violence due to the drug trade, and conflict-related poverty, all created increased risks for women of exposure to IPV.

In their qualitative comparative analysis of women’s experience of IPV in Northern Ireland from conflict to post-conflict, Doyle and McWilliams (2020) identified a significant role of policing and access to justice. The study examined the ways in which the occurrence of violent conflict can affect police responsiveness to IPV and protection of victims. Doyle and McWilliams (2020) noted that during the conflict in Northern Ireland, Catholic or nationalist communities often viewed the police as a source of harassment or discrimination, rather than protection, which further reduced victims’ likelihood of seeking access to justice by these means. In this context, paramilitarism also impacted women’s experiences of IPV. Respondents interviewed by Doyle and McWilliams (2020) described how impunity, power and control associated with paramilitary membership continued to influence IPV in Northern Ireland many years after the official cessation of the political conflict, and that paramilitary involvement (whether genuine or fabricated) could provide an additional source of power for perpetrators to leverage over victims.

Availability of firearms was highlighted by Doyle and McWilliams (2020) as a factor that affected women’s experiences of IPV in Northern Ireland. This was experienced by the women respondents as an additional source of threat that perpetrators exploited. This is similar to findings reported by Gerlock et al. (2014) and Friedman et al. (2025) of US Army veterans and their partners, where partners’ knowledge of the veterans’ capacity to access weapons was seen as a factor that instigated significant fear and anxiety among partners.

## Refugee and internally displaced communities

Several studies specifically examined drivers of the relationship between armed conflict and IPV in the context of migration and displacement. These samples included Syrian refugee women in Jordan (Daoud, 2021; Al-Natour et al., 2022), Afghan women in the UK (Azizi et al., 2024), women refugees from Burundi and the DRC in Uganda (Lukasiak et al., 2024), Venezuelan women in Brazil (Makuch et al., 2021), refugee communities in Kenya, Iraq and South Sudan (Wachter et al., 2018), and communities of internally displaced persons in Columbia (Wirtz et al., 2014) and Côte d’Ivoire (Cardoso et al., 2016). Similar to studies carried out across other samples and contexts, mental distress associated with war and armed conflict was commonly cited as a driver of IPV. For example, in a qualitative study of Syrian refugee women living in Jordan, Al-Natour et al. (2022) highlighted men’s war-related mental health problems as a factor perceived by women to affect IPV perpetration. Respondents reported how their husbands became violent for the first time during the war. One participant stated: ‘The war emotionally affected my husband a lot. His nervousness increased a lot here in Jordan. He became extreme, now he has a temper, he is so moody and a troublemaker, if something bothered him in his work, he starts fighting with us for no reason’, (Al-Natour et al., 2022, p. 28,).

Similar to the studies from conflict-affected contexts, studies with refugee and IDP communities also highlighted the significance of economic factors in increasing IPV risk in conflict-affected settings. For example, in Wachter et al.’s (2018) study of refugee samples in South Sudan, Kenya and Iraq, it was reported that economic deprivation in camp settings in some cases increased the likelihood of women marrying rapidly or being forced into marriage, which was a factor that could also increase the risk of IPV. The same dynamic was reported by Daoud (2021) in a study of Syrian refugees living in Jordan.

As noted in relation to research from conflict-affected countries, gender dynamics also emerged as significant in relation to IPV in refugee and IDP communities. Included studies described various ways in which conflict and displacement affect gender norms in ways that can, in turn, affect the occurrence of IPV. For example, Wachter et al. (2018) found that women in some refugee camps reported that opportunities to attend training and develop skills resulted in empowerment, which was threatening to men and could precipitate IPV. Some respondents reported that IPV was perpetrated due to men’s perception that women were adopting Western cultural values. Similarly, in a qualitative study with Liberian refugee women living in South Australia, Zannettino (2012) reported that cultural differences between Australia and Liberia were perceived as increasing the risk of IPV, whereby men were reported as sometimes perpetrating IPV due to feeling threatened by women’s increased autonomy in the new context. On the other hand, some respondents interviewed by Wachter et al. (2018), who were migrating from more liberal settings, reported finding the camps to be oppressive and highly conservative, feeling their freedoms were limited and their experience of harassment and judgement intensified. The stress of migration was also highlighted in some included studies as affecting the risk of IPV. For example, respondents in Cardoso et al. (2016) described how social discrimination against internally displaced people (IDPs) resulted in greater vulnerability to poverty, food insecurity and IPV for women. In a study of Venezuelan refugee women in Brazil (Makuch et al., 2021), xenophobia from the local community was described as compounding other forms of violence they experienced, including violence from their partners.

In a study across three refugee camps Kenya (Dadaab), South Sudan (Ajuong Thok) and Iraq (Domiz) by Wachter et al. (2018), some respondents reported that men using alcohol or other substances to cope with the stress of camp life and unemployment increased the risk of IPV. Wachter et al. (2018) also noted that displacement acted as a risk factor for IPV by removing people from their social support and protective networks. In a study of Afghan women in the UK seeking asylum or family reunification (Azizi et al., 2024), women reported experiences of multiple types of violence, including pre-migration political violence, IPV and other types of family violence. Azizi et al., 2024 noted how migration to flee from conflict and violence can create increased vulnerability to experiencing IPV due to language barriers in the host country, lack of knowledge about criminal justice systems, and financial dependency on the perpetrator. In a study of Syrian refugee women in Jordan (Al-Natour et al., 2022), respondents perceived that economic instability and uncertainty were exacerbated by relocation to a country with a higher cost of living, which in turn created a higher risk of IPV perpetration.

Other migration-related stressors highlighted by Liberian refugee women living in Australia (Zannettino, 2012) included coping with the experience of having lived through conflict, experiencing conflict-related violence, needing to flee, the grief of losing family members to violence or of needing to leave family members behind, as well as the stress associated with living in transient accommodation. Zannettino’s (2012) findings highlight how migration-related stressors create emotional dysregulation and gender role stress that may increase the likelihood of IPV.

## Discussion

This review examined the state of current evidence on drivers of the relationship between armed conflict and IPV. A range of individual, relational and societal drivers of the relationship were identified. Among the most-studied factors were psychological trauma, PTSD and related social and mental health problems. Several included studies noted the significant role of trauma and PTSD, including exposure to the most extreme forms of conflict-related violence, such as experiencing atrocities (e.g., Taft et al., 2005) or torture (Rees et al., 2018). This is consistent with existing literature on the impact of trauma (e.g., May and Wisco, 2016), which shows that greater severity and proximity are associated with poorer post-trauma outcomes. Included studies explored various ways in which trauma and PTSD could increase the risk of perpetration, including via problematic use of alcohol and other drugs (Creech et al., 2017; Iverson et al., 2020; Mannell et al., 2021a). The findings in relation to the impact of substance use, particularly alcohol, are also consistent with the wider research on IPV, supporting a significant association between alcohol and increased risk of perpetration (Foran and O’Leary, 2008).

Also similar to the wider literature on IPV risk factors, the review identified significant overlap between the factors that increase the risk of perpetration and those that increase the risk of victimisation. Several included studies examined the impact of conflict-related trauma and its related emotional dysregulation difficulties on both perpetration and victimisation. Two studies (Saile et al., 2013; Sileo et al., 2021) found that trauma in women and related re-experiencing (Saile et al., 2013) and attachment difficulties (Sileo et al., 2021) may increase vulnerability to experiencing IPV. Although the cross-sectional design of these studies means that the temporal relationship between the variables is not certain, the finding does appear consistent with the wider research on psychological trauma suggesting that experiencing one type of trauma or adverse experience increases the risk of experiencing others, and that those who experience multiple types of adversities are at the highest risk of experiencing more serious and enduring consequences (Felitti et al., 1998; Finkelhor et al., 2007). The included qualitative studies also provide insights into the nuances of how trauma can act as a vulnerability factor for victimisation. For example, several studies (e.g., Kelly et al., 2012; Kiconco and Nthakomwa, 2018; Daoud, 2021) described how shame and stigma placed on women who had experienced conflict-related sexual violence can be used as a justification for further abuse perpetrated against them by partners, families and the wider community.

However, it should be noted that most of the information on trauma as a risk factor comes from relatively homogenous samples, with a disproportionate number of studies conducted with US Army veterans. This is likely to be partly a product of global research funding inequalities. However, it may also be reflective of cultural and contextual differences in how researchers from different countries tend to perceive the impact of war and armed conflict. Military personnel from high-income countries experience armed conflict differently from people who live daily with the impact of war and violence in their communities over long periods of time. This likely plays a role in the tendency of research with samples based in high-income countries such as the US, UK and Israel to consider the impact of war in terms of more individual-level problems of clinical diagnoses of PTSD (e.g., Orcutt et al., 2003; Taft et al., 2005; Taft et al., 2007), for example, or of ‘aggressive impulses’ (Snir et al., 2017). On the other hand, research conducted in LMICs tended to consider the broader impacts of war and its destruction of the social and economic fabric of communities and societies, as well as its individual mental health impacts (e.g., Falb et al., 2014; Daoud, 2021; Al-Natour et al., 2022). The analysis presented in this review, therefore, highlights some of the ways in which exposure to the ongoing stress, trauma and instability of a post-conflict context, or being forced to flee a conflict-affected setting and experiencing migratory stresses (e.g., Cardoso et al., 2016), differs from the experience of being exposed to conflict and then returning to high-income countries. Of the 24 quantitative studies included, the majority were based on US Army veterans and their partners. Only four quantitative studies based in Africa (Gupta et al., 2012; Saile et al., 2013; Sileo et al., 2021; Bourey et al., 2024) that met the study inclusion criteria were identified, despite Africa being one of the most conflict-affected regions in the world. This review, therefore, indicates a need for more geographically diverse research to examine how contextual factors interact with individual-level variables.

Some relationship-level factors were identified by included studies as being significantly associated with IPV. For example, Gerlock et al. (2016) identified ‘relationship mutuality’ as a factor that distinguished IPV perpetrators from non-perpetrators. However, it appears that there may be a possibility of some conceptual overlap between the variable of ‘relationship mutuality’ and relationship quality, which may produce a tautological analysis when used as a predictor of IPV. A similar potential problem presents in relation to the interpretation of findings by Creech et al. (2017), which identified that relationship satisfaction explained significant variance in psychological IPV. It seems that it would stand to reason that dissatisfaction would be more likely in a relationship where psychological abuse is present, and the directionality of the relationship here is unclear. Longitudinal studies are needed to understand which relationship factors may act as early predictors of IPV, and how these factors in turn may be related to conflict exposure.

The findings of this review highlight the importance of an intersectional approach to understanding and preventing violence in conflict-affected settings. Intersectionality brings a focus on the role of power relations in contributing to partner abuse, and on the relationship between structural and interpersonal forms of domination and control (Collins, 2024). For example, some included studies (e.g., Gerlock et al., 2014; Jewkes et al., 2017) examined how newly acquired disabilities from conflict exposure could negatively impact intimate relationships, through changing the relationship dynamic such that one partner must now act as a carer for the other. This change in power dynamics can create increased vulnerability to partner abuse. In their description of their sampling procedure, which drew on Veteran Health Administration screening centres, Iverson et al. (2020) noted that US Army personnel are routinely screened for traumatic brain injury (TBI), and that 20% screen positive. Experiences of people with different types of disabilities acquired as a result of war and armed conflict are an area that warrants further consideration in research and humanitarian programming.

The intersection between migration status and gender was also considered in many of the included studies. For example, a study by Azizi et al. (2024) highlighted how migration-related vulnerabilities, such as language barriers and a lack of knowledge about local services, can be used by perpetrators to exert greater control over victims. However, these intersections between migration and gender issues, including gender-based violence, are often under-considered in law, policy and practice (Tastsoglou and Nourpanah, 2019). Additionally, despite the broader consideration of the impacts of war and armed conflict presented in studies conducted in conflict-affected and displacement contexts, it was still uncommon for these studies to engage with the legacy of colonialism as a factor shaping the conditions for armed conflict and gendered violence to occur. All of the conflict-affected contexts considered in the studies included in the review had experienced colonial rule or imperial domination at some point in their histories. However, this history was rarely acknowledged; where colonialism was mentioned, it was primarily in a descriptive sense – providing background information to the study – rather than being explicitly theorised as a structural force relevant to understanding ongoing violence and instability. While theory-building in this area does consider structural influences, including the political and economic context (e.g., Cockburn et al., 2004; True, 2010) and broader dynamics such as globalisation (Murphy et al., 2023), it often stops short of theorising the impact of colonialism on these structures. Given the close links between colonial violence, instability, armed conflict and gendered violence (e.g., Lugones, 2007; Smith, 2015; Segato and Monque, 2021; Mannell et al., 2021b), closer engagement with these historical and structural dynamics appears important to developing a fuller understanding of how armed conflict perpetuates IPV.

### Limitations

This review provides an overview of the current research evidence on the drivers of the association between armed conflict and IPV. However, it is not without limitations. Due to the fact that the majority of the included literature is cross-sectional, the directionality of the relationships explored cannot be assumed. We also acknowledge the limitations inherent in considering ‘armed conflict’ as an exposure, which fails to capture the diversity and complexities of individual experiences, and the socio-political context in which it occurs. Future research and theory-building may identify preferable ways to measure and conceptualise these experiences. Several studies were excluded from consideration from this review due to being based on samples of women of reproductive age (15–49), and not disaggregating analyses between adolescents and adult women. This may have resulted in some potentially relevant findings being overlooked. However, we propose that this also indicates a need for purpose-designed research investigating this topic to better elucidate the causal mechanisms of the relationship. The practice of only considering partner violence against women aged 15–49 obfuscates issues specifically affecting adolescents, as well as violence experienced by middle-aged and older women.

### Conclusions and future perspectives

Despite the clear associations between armed conflict and IPV, recognition of this intersection in policy and practice has been relatively slow. More research is needed to explore the ways in which IPV in conflict-affected contexts can best be addressed through intervention. However, the studies included in this review also present some examples of promising practices with potential for scaling to other contexts. For example, Doyle and McWilliams (2020) have highlighted the use of community-based restorative justice (CBRJ) schemes in Northern Ireland. These schemes involve non-violent resolution of crimes, usually through mediation, as an alternative to paramilitary involvement in communities where trust in police is low. Although these schemes to date have not usually been oriented towards IPV, there may be potential to explore a more significant role for such initiatives in IPV prevention, particularly in post-conflict contexts. As noted by Doyle and McWilliams (2020), they may present greater opportunities for community identification, ownership, acceptability and sustainability.

In terms of future research and theory-building, studies including mediation analyses will be useful to better understand the processes by which the factors identified in this review act to increase the risk of IPV. Studies gathering data at more than one time point are also needed to clarify the temporality of the associations identified. Additionally, most included quantitative studies tended to focus on individual-level risk factors for IPV, whereas the qualitative studies tended to highlight the role of structural and social factors. However, as outlined already, and consistent with an intersectional perspective (e.g., Bowleg and Bauer, 2016; Collins, 2024), researchers should endeavour to integrate risk factors operating at multiple levels to produce a more complete understanding of this association.

## Supporting information

10.1017/gmh.2026.10248.sm001 Travers et al. supplementary material Travers et al. supplementary material

## Data Availability

As this was a systematic review, there is no data, therefore this section is not applicable.

## Supplementary material

The supplementary material for this article can be found at http://doi.org/10.1017/gmh.2026.10248.

## Long descriptions

### Figure 1. Long description

The flowchart is divided into three vertical sections: Identification, Screening, and Included.

1. Identification Phase:

* Top-left box: Studies from databases/registers n = 5636. Breakdown: Psyc I N F O n = 1996, PubMed n = 1511, Web of Science n = 1381, C I N A H L n = 421, Embase n = 327.

* Top-right box: References from other sources n = 4.

* These two boxes merge into a central line. A lateral arrow points to a box: References removed n = 1290. Breakdown: Duplicates identified manually n = 23, Duplicates identified by Covidence n = 1267.

2. Screening Phase:

* Central box: Studies screened n = 4350. An arrow points right to: Studies excluded n = 4115.

* Central box: Studies sought for retrieval n = 235. An arrow points right to: Studies not retrieved n = 0.

* Central box: Studies assessed for eligibility n = 235. An arrow points right to a detailed exclusion box: Studies excluded n = 186. Breakdown: Wrong I V n = 17, Wrong sample n = 66, Wrong setting n = 5, Wrong outcomes n = 43, Wrong study design n = 45, Unpublished dissertations n = 1, Reviews, commentaries etc. n = 9.

3. Included Phase:

* Final central box at the bottom: Studies included in review n = 49.

Navigate back to Figure 1..

### Table 1. Long description

The table consists of 25 rows, each detailing a specific research study.

* Al-Natour et al. 2022, Jordan: Studied 16 Syrian refugee women using content analysis. Found war-related financial stress and changed husband behaviors led to increased I P V.

* Azizi et al. 2024, United Kingdom: Studied 15 Afghan women using thematic analysis. Found patriarchal power and lack of rights awareness increased dependence and violence risk.

* Cardoso et al. 2016, Cote d'Ivoire: Focus groups with 91 participants using grounded theory. Found loss of livelihoods and changed gender roles increased I P V risk.

* Daoud 2021, Jordan: Narrative interviews with 23 Syrian women and 13 providers. Found interconnected risks including disruption of cultural codes and conflict-related trauma.

* Doyle and McWilliams 2020, Northern Ireland: Comparative interviews from 1992 and 2016. Found transition to peace improved reporting, but paramilitary influence on control persisted.

* Falb et al. 2014, Cote d'Ivoire: Interviews with 32 men. Found economic empowerment programs for women were accepted but challenged traditional male provider identities.

* Falb et al. 2022, Myanmar and D R C: Interviews and focus groups with over 100 participants. Found power imbalances and exposure to armed conflict exacerbated domestic violence.

* Finley et al. 2010, United States: Interviews with 19 male veterans and 11 spouses. Categorized I P V into anger-based, dissociative, and parasomniac violence related to P T S D.

* Fitzgerald et al. 2021, Palestine: Interviews with 25 women in Gaza. Identified societal expectations and distrust of services as barriers to help-seeking.

* Friedman et al. 2025, United States: Secondary analysis of military partner interviews. Found high firearm ownership and alcohol use as significant risk factors.

* Gerlock et al. 2014, United States: Study of 441 veteran couples. Found P T S D and deployment-related disabilities added relationship stress and increased vulnerability to abuse.

* Guruge et al. 2017, Sri Lanka: Interviews with 15 survivors and 15 providers. Found I P V related to war trauma and men using violence to regain perceived lost masculinity.

* Horn et al. 2014, Sierra Leone and Liberia: Focus groups and interviews with 130 women. Found war normalized violence but sometimes increased women's economic independence.

* Kaul et al. 2024, Afghanistan: Interviews with 60 women and 60 men. Found compounding traumatic experiences of war and lack of mental health services worsened outcomes.

* Kattoura 2022, Israel: Interviews with 36 Arab women. Found structural oppression and political violence projected inward into private intimate relationships.

* Kelly et al. 2012, D R C: Focus groups with 86 participants. Found I P V often resulted from a cycle of blame and anger following conflict-related sexual violence.

* Kiconco and Nthakomwa 2018, Uganda: Interviews with 40 ex-abductees. Found social stigma against women abducted by the L R A reduced reintegration opportunities.

* Kohli et al. 2015, D R C: Interviews with 13 survivors and 5 perpetrators. Linked I P V to alcohol use, economic instability, and gendered power dynamics.

* Lukasiak et al. 2024, Uganda: Interviews with 13 refugee women. Found shifting power dynamics in camps where women became providers increased violence risk.

* Makuch et al. 2021, Brazil: Focus groups with 111 Venezuelan migrant women. Found physical and psychological violence common in shelters, driven by rigid gender roles.

* Mannell et al. 2021a, Afghanistan: Interviews with 20 women. Linked domestic violence to the loss of patriarchal support and the conflict-driven drug trade.

* Restrepo et al. 2024, Colombia: Interviews with 47 women. Found armed conflict amplified patriarchal norms and hypermasculinity.

* Wachter et al. 2018, South Sudan, Kenya, and Iraq: 284 participants. Found displacement destabilized gender roles and increased risk through forced or rapid marriages.

* Wirtz et al. 2014, Colombia: Interviews with 35 survivors and 31 providers. Found G B V perpetrated by both armed actors and intimate partners during displacement.

* Zannettino 2012, Australia: Focus groups with Liberian refugee women. Found migration stressors and changing gender norms made men feel threatened by women's autonomy.

Navigate back to Table 1..

### Table 2. Long description

The table consists of 8 columns: Authors, year, country; Sample details; Reported I P V; Study design; Analysis; Study aims/objectives; Main findings; and Proposed driver.

Key entries include:

* Botchkovar et al. 2025, Ukraine: Random sample of 1,185 adults. Findings show daily stressors, negative emotions, and alcohol use predicted I P V, while war exposure had an indirect effect.

* Bourey et al. 2024, Democratic Republic of Congo: 2,080 men. Findings linked interpersonal violence and trauma symptoms to I P V use.

* Cesur and Sabia 2016, United States: 11,474 service men. Combat exposure was associated with domestic violence, mediated by mental health and substance use.

* Creech et al. 2017, United States: 102 women veterans. Alcohol issues and psychological I P V experience were associated with physical I P V use.

* Chiu et al. 2022, United States: 217 veterans. T B I and neurocognitive performance predicted subsequent I P V.

* Gibbs et al. 2021, Palestine: 534 women. Occupation-related events increased I P V experience via depressive symptoms and relationship conflict.

* Gupta et al. 2012, South Africa: 772 men. Significant association between human rights violations and physical I P V perpetration.

* Jewkes et al. 2017, Papua New Guinea: 746 men and 793 women. War trauma related to P T S D and alcohol problems, which affected I P V perpetration.

* Lane et al. 2022, United Kingdom: 4,566 military personnel. Risk factors included childhood adversity, military trauma, and mental health problems.

* Rees et al. 2018, Timor-Leste: 870 individuals. Torture exposure directly and indirectly (via P T S D and drinking) associated with I P V.

* Rojczyk et al. 2024, United States: 49 veterans. Neuroimaging showed psychological aggression associated with right amygdala-hippocampus complex abnormalities.

* Taft et al. 2007, 2015, United States: Multiple studies linking P T S D, hyperarousal, and hostile attribution biases to I P V perpetration.

Navigate back to Table 2..

### Table 3. Long description

The table consists of four columns and six rows of data.

1. Trauma, mental health and emotion regulation problems: Military samples list P T S D, depression, anger, T B I, and neurocognitive problems. Conflict-affected contexts list trauma symptoms and psychological distress. Refugee and I D P communities list war-related mental health problems and migration-related stress.

2. Substance use: All three populations list problematic alcohol and substance use, with conflict-affected contexts also noting drug trade issues.

3. Relationship: Military samples list relationship satisfaction, emotional intimacy, mutuality, and changing dynamics with disability. Conflict-affected contexts have no data. Refugee and I D P communities list forced or early marriage.

4. Economic factors: Military samples have no data. Conflict-affected contexts list poverty, financial stress, and livelihood loss. Refugee and I D P communities list camp deprivation and lack of economic opportunity.

5. Gender dynamics: Military samples have no data. Conflict-affected contexts list gender role shifts, threats to the provider role, and stigma of conflict-related sexual violence. Refugee and I D P communities list men threatened by women's autonomy and adoption of new values.

6. Social and structural factors: Military samples list access to weapons. Conflict-affected contexts list policing issues, paramilitarism, loss of social support, and access to weapons. Refugee and I D P communities list discrimination, language barriers, loss of social support, and overcrowding in camps.

Navigate back to Table 3..
