# Supplementary material for: Drivers of the association between armed conflict and intimate partner violence: A systematic review
Source: Glob Ment Health (Camb). 2026 Jun 15;13:e126. doi: 10.1017/gmh.2026.10248 (PMC13312369; doi:10.1017/gmh.2026.10248)
Supplement: Travers et al. supplementary material [file S2054425126102489sup001.zip › Supp_file1_Search strategy.docx]

**Web of Science**

Search in Topic

(humanitarian OR war OR wars OR warzone* OR unrest OR militant* OR "post conflict" OR post-conflict OR refugee* OR displacement OR "displaced persons" OR ((armed OR political) NEAR/2 (conflict* OR violence OR attack*)))

AND

("intimate partner violence" OR IPV OR "partner violence" OR "domestic violence" OR "domestic abuse" OR "partner abuse" OR "spouse abuse" OR "spousal abuse" OR "spousal violence" OR "dating abuse" OR "dating violence" OR "family violence")

**EMBASE**

('war'/exp OR 'migrant'/exp OR humanitarian:ab,ti,kw OR war:ab,ti,kw OR wars:ab,ti,kw OR warzone*:ab,ti,kw OR militant*:ab,ti,kw OR "post conflict":ab,ti,kw OR refugee*:ab,ti,kw OR "displaced persons":ab,ti,kw OR ((armed OR political) NEAR/2 (conflict* OR violence OR attack*)):ab,ti,kw ) NOT [MEDLINE]/lim AND [english]/lim

AND

('IPV'/exp OR "intimate partner violence":ab,ti,kw OR "partner violence":ab,ti,kw OR "domestic violence":ab,ti,kw OR "domestic abuse":ab,ti,kw OR "partner abuse":ab,ti,kw OR "spouse abuse":ab,ti,kw OR "spousal abuse":ab,ti,kw OR "spousal violence":ab,ti,kw OR "dating abuse":ab,ti,kw OR "dating violence":ab,ti,kw OR "family violence":ab,ti,kw) NOT [MEDLINE]/lim AND [english]/lim

**CINAHL**

(MH "War+" OR MH "Transients and Migrants" OR MH "Refugees+" OR TI(humanitarian OR war OR wars OR warzone* OR militant* OR "post conflict" OR "post-conflict" OR refugee* OR "displaced persons" OR ((armed OR political) N2 (conflict* OR violence OR attack*))) OR AB (humanitarian OR war OR wars OR warzone* OR militant* OR "post conflict" OR "post-conflict" OR refugee* OR "displaced persons" OR ((armed OR political) N2 (conflict* OR violence OR attack*))))

AND

(MH "Intimate Partner Violence+" OR TI("intimate partner violence" OR "partner violence" OR "domestic violence" OR "domestic abuse" OR "partner abuse" OR "spouse abuse" OR "spousal abuse" OR "spousal violence" OR "dating abuse" OR "dating violence" OR "family violence") OR AB("intimate partner violence" OR "partner violence" OR "domestic violence" OR "domestic abuse" OR "partner abuse" OR "spouse abuse" OR "spousal abuse" OR "spousal violence" OR "dating abuse" OR "dating violence" OR "family violence")

**PSYCINFO**

(DE "War" OR DE "Refugees" OR DE "Political Violence" OR DE "Armed Conflict" OR TI(humanitarian OR war OR wars OR warzone* OR militant* OR "post conflict" OR "post-conflict" OR refugee* OR survivor* OR "displaced persons" OR ((armed OR political) N2 (conflict* OR violence OR attack*))) OR AB(humanitarian OR war OR wars OR warzone* OR militant* OR "post conflict" OR "post-conflict" OR refugee* OR survivor* OR "displaced persons" OR ((armed OR political) N2 (conflict* OR violence OR attack*))))

AND

(DE "intimate partner violence" OR TI("intimate partner violence" OR IPV OR "partner violence" OR "domestic violence" OR "domestic abuse" OR "partner abuse" OR "spouse abuse" OR "spousal abuse" OR "spousal violence" OR "dating abuse" OR "dating violence" OR "family violence") OR AB("intimate partner violence" OR IPV OR "partner violence" OR "domestic violence" OR "domestic abuse" OR "partner abuse" OR "spouse abuse" OR "spousal abuse" OR "spousal violence" OR "dating abuse" OR "dating violence" OR "family violence"))

**PubMed**

(War[Mesh] OR "Armed Conflicts"[Mesh] OR "Political Violence"[Mesh] OR Refugees[Mesh] OR "Displaced Persons"[Mesh] OR "Humanitarian Emergencies"[Mesh] OR humanitarian[tiab] OR war[tiab] OR wars[tiab] OR warzone*[tiab] OR militant*[tiab] OR "post conflict"[tiab] OR "post-conflict"[tiab] OR refugee*[tiab] OR displacement[tiab] OR "displaced persons"[tiab] OR ((armed OR political) AND (conflict*[tiab] OR violence[tiab] OR attack*[tiab])))

AND

("Intimate Partner Violence"[Mesh] OR "Domestic Violence"[Mesh] OR "Spouse Abuse"[Mesh] OR "intimate partner violence"[tiab] OR IPV[tiab] OR "partner violence"[tiab] OR "domestic violence"[tiab] OR "domestic abuse"[tiab] OR "partner abuse"[tiab] OR "spouse abuse"[tiab] OR "spousal abuse"[tiab] OR "spousal violence"[tiab] OR "dating abuse"[tiab] OR "dating violence"[tiab] OR "family violence"[tiab])
