# Supplementary material for: Drivers of the association between armed conflict and intimate partner violence: A systematic review
Source: Glob Ment Health (Camb). 2026 Jun 15;13:e126. doi: 10.1017/gmh.2026.10248 (PMC13312369; doi:10.1017/gmh.2026.10248)
Supplement: Travers et al. supplementary material [file S2054425126102489sup001.zip › Supp_file2_MMAT for qualitative studies.docx]

MMAT for qualitative studies

|  | Clear research questions | Data allows research questions to be answered | Qualitative approach appropriate | Qualitative data collection methods adequate to address the research question | Findings adequately derived from the data | Interpretation of results sufficiently substantiated by data | Coherence between qualitative data sources, collection, analysis and interpretation |
| --- | --- | --- | --- | --- | --- | --- | --- |
| Al-Natour et al. (2022) | Yes | Yes | Yes | Yes | Yes | Yes | Yes |
| Azizi et al. (2023) | Yes | Yes | Yes | Yes | Yes | Yes | Yes |
| Cardoso et al. (2016) | Yes | Yes | Yes | Yes | Yes | Yes | Yes |
| Daoud (2021) | Yes | Yes | Yes | Yes | Yes | Yes | Yes |
| Doyle & McWilliams (2020) | Yes | Yes | Yes | Yes | Yes | Yes | Yes |
| Falb et al. (2014) | Yes | Yes | Yes | Yes | Yes | Yes | Yes |
| Falb et al. (2022) | Yes | Yes | Yes | Yes | Yes | Yes | Yes |
| Finley et al., (2010) | Yes | Yes | Can't tell  Insufficient detail on analytic approach. | Can't tell  Findings based on case studies of interviews initially gathered for another purpose. Process of selecting cases unclear. | Can't tell  Insufficient detail on analytic processes. | Yes | Can't tell  Insufficient detail on analytic approach. |
| Fitzgerald et al. (2021), | Yes | Yes | Yes | Yes | Yes | Yes | Yes |
| Friedman et al. (2025) | Yes | Yes | Yes | Yes | Yes | Yes | Yes |
| Gerlock et al.,(2014) | Yes | Yes | Yes | Yes | Yes | Yes | Yes |
| Guruge et al. (2017) | Yes | Yes | Yes | Yes | Yes | Yes | Yes |
| Horn et al. (2014) | Yes | Yes | Yes | Yes | Yes | Yes | Yes |
| Kaul et al. (2024) | Yes | Yes | Yes | Yes | Yes | Yes | Yes |
| Kattoura (2022), | Yes | Yes | Yes | Can't tell  Study seeks to explore how historical and political processes shape men’s attitudes toward honour and women’s entrapment; however, data were collected solely from women. | Can't tell  Insufficient detail on analytic processes. | Can't tell  Analytic pathway between discourse patterns and psychoanalytic conclusions about society unclear. | Can't tell  Extension of interpretation to societal psychoanalytic processes not clearly outlined, limiting assessment of coherence between analytic approach and interpretation. |
| Kelly et al. (2012) | Yes | Yes | Yes | Yes | Yes | Yes | Yes |
| Kiconco & Nthakomwa. (2018), | Yes | Yes | Can't tell  Insufficient detail on analytic approach. | Yes | Can't tell  Insufficient detail on analytic processes. | Yes | Can't tell.  Insufficient detail on analytic approach. |
| Kohli et al. (2015), | Yes | Yes | Yes | Yes | Yes | Yes | Yes |
| Lukasiak et al. (2024). | Yes | Yes | Yes | Yes | Yes | Yes | Yes |
| Makuch et al. (2021) | Yes | Yes | Yes | Yes | Yes | Yes | Yes |
| Mannell et al. (2021) | Yes | Yes | Yes | Yes | Yes | Yes | Yes |
| Restrepo et al. (2024). | Yes | Yes | Yes | Yes | Yes | Yes | Yes |
| Wachter et al. (2018) | Yes | Yes | Yes | Yes | Yes | Yes | Yes |
| Wirtz et al., (2014) | Yes | Yes | Yes | Yes | Yes | Yes | Yes |
| Zannettino (2012) | Yes | Yes | Yes | Yes | Yes | Yes | Yes |
