# Supplementary material for: Drivers of the association between armed conflict and intimate partner violence: A systematic review
Source: Glob Ment Health (Camb). 2026 Jun 15;13:e126. doi: 10.1017/gmh.2026.10248 (PMC13312369; doi:10.1017/gmh.2026.10248)
Supplement: Travers et al. supplementary material [file S2054425126102489sup001.zip › Supp_file3_MMAT for quant studies.docx]

MMAT for quantitative studies

|  | Clear research questions | Data allows research questions to be answered | Participants representative of target population | Measurements appropriate | Complete outcome data | Confounders accounted for | Intervention administered (or exposure occurred) as intended |
| --- | --- | --- | --- | --- | --- | --- | --- |
| Botchkovar et al. (2025) | Yes | Yes | Yes | Yes | Yes | Yes | Yes |
| Bourey et al. (2024) | Yes | Yes | No  Villages selected purposively. | Yes | Yes | Yes | Yes |
| Cesur & Sabia (2016) | Yes | Yes | Yes | Yes | Can't tell  Significant proportion of cases excluded due to non-missing and other DV information. Specific proportion missing due to DV not reported. | Yes | Yes |
| Chiu et al. (2022) | Yes | Yes | No  Participants recruited from an eligible subset of a wider cohort and restricted to cohabiting couples who consented to participate. | Yes | No  Significant proportion of missingness (approx. 20%) | Yes | No  Deployment to a combat zone was an eligibility criterion rather than a measured exposure variable. |
| Creech et al. (2017) | Yes | Yes | No  Despite random selection, low response rate (27%) and restriction to respondents in current relationships limits representativeness. | Yes | Yes | No  Regression models did not adjust for demographic confounders (e.g., age, education), and covariate inclusion was based on bivariate significance. | Yes |
| Gerlock et al. (2016) | Yes | Yes | No  Some eligibility criteria (e.g., being in current heterosexual relationships, partner consent) limits representativeness of broader population. | Yes | Yes | No  Analyses were limited to bivariate correlations and group comparisons; no multivariable models were used to adjust for potential confounders. | No  Armed conflict exposure was inferred from clinical PTSD treatment context rather than directly measured or operationalised. |
| Gibbs et al. (2021) | Yes | Yes | Yes | Yes | Yes | Yes | Yes |
| Gupta et al. (2012) | Yes | Yes | Yes | Yes | No  Sample size drops significantly in adjusted analyses due to missing covariate data. | Yes | Yes |
| Heavey et al., (2017) | Yes | Yes | No  Recruitment via military drills plus eligibility criteria (partner participation, alcohol use etc.). | Yes | Yes | Yes | Yes |
| Iverson et al. (2020) | Yes | Yes | No  Low response rate and systematic differences identified between responders and non-responders. | Yes | No  Low initial response rate and 20% of female respondents did not complete IPV items. | No  Analyses were limited to bivariate comparisons; no multivariable models were used to adjust for demographic differences. | No  Deployment to conflict zone was an eligibility criterion; variation or intensity of combat exposure not analysed. |
| Jewkes et al. (2017) | Yes | Yes | Yes | Yes | Yes | Yes | Yes |
| Kar & O’Leary (2013) | Yes | Yes | No  Sample drawn from single site, plus eligibility criteria limit representativeness. | Yes | Yes | No  Authors report that demographic covariates not included due to limited sample size. | No  Deployment to conflict zone was an eligibility criterion; variation or intensity of combat exposure not analysed. |
| Lane et al. (2022) | Yes | Yes | Yes | Yes | Yes | Yes | Yes |
| Orcutt et al. (2003) | Yes | Yes | No  Non-random family sub-study from a wider national study. | Yes | Yes | Structural equation model included developmental and war-related variables only, no demographic covariates were included. | Yes |
| Rees et al. (2018) | Yes | Yes | Yes | Yes | Yes | Yes | Yes |
| Rojczyk et al. (2024) | Yes | Yes | No  Single site clinic recruitment with small final sample. | Yes | No  Only 49 of 384 recruited veterans had complete IPV and neuroimaging data but no information on reasons for missingness available. | No  No comprehensive adjustment for demographic confounders beyond age. | Yes |
| Saile et al. (2013) | Yes | Yes | No  Purposive sampling of communities. | Yes | Yes | Yes | Yes |
| Sileo et al. (2021) | Yes | Yes | No  Single site recruitment plus eligibility criteria limit representativeness. | Yes | Yes | Yes | Yes |
| Snir et al. (2017) | Yes | Yes | No  Follow-up cohort with attrition and no clear probability sampling limits representativeness. | Yes | Yes | Yes | No  Conflict exposure inferred via ex-combatant status rather than directly measured. |
| Taft et al. (2005) | Yes | Yes | Yes | Yes | Yes | No  No multivariable model adjusting for demographic confounders. | Yes |
| Taft et al. (2007) | Yes | Yes | No  Single site convenience sample, plus eligibility restrictions. | Yes | Yes | No  Exploratory ANCOVAs included income as a covariate but no systematic multivariable adjustment for demographic confounders in main analyses. | Yes |
| Taft et al. (2015) | Yes | Yes | No  Single-site volunteer sample with noted differences from wider veteran population on characteristics including age and racial diversity. | Yes | Yes | No  Mediation models did not include demographic covariates - analyses appear unadjusted. | Yes |
| Teten et al. (2010) | Yes | Yes | No  Single site recruitment with high non-response rate. | Yes | Yes | No  Authors state that limited sample size precluded inclusion of demographic covariates. | Yes |
| Tharp et al. (2016) | Yes | Yes | No  Clinic-based convenience sample with exclusion criteria e.g., severe IPV and alcohol use. | Yes | Yes | No  No systematic adjustment for potential demographic covariates. | No  Combat exposure inferred through sample characteristics, not directly modelled. |
