# Supplementary material for: Drivers of the association between armed conflict and intimate partner violence: A systematic review
Source: Glob Ment Health (Camb). 2026 Jun 15;13:e126. doi: 10.1017/gmh.2026.10248 (PMC13312369; doi:10.1017/gmh.2026.10248)
Supplement: Peer review history

# Reviewer report: Review: Drivers of the association between armed conflict and intimate partner violence: A systematic review — R0/PR2

**DOI:** 10.1017/gmh.2026.10248.pr2

Background

I know you won’t have the space to mention all research on this topic - but feels like you are missing some seminal work on the topic - such as Ellsberg’s work in South Sudan. There also was a recent systematic review and meta analysis on IPV in conflict (https://pubmed.ncbi.nlm.nih.gov/39717938/) that may be helpful to cite.

You also seem to be referring to continuums theory in your comment about violence continuing in a private sphere after a conflict- but don’t cite Cockburn, True, Swaine, etc. who do work on these concepts.

More recent reviews of risk and protective factors for VAWG and IPV in conflict settings are also available - so its odd to focus on research from 2010 as your example of a growing evidence base.

Methods

It is a bit strange to have a public heath review without using Pubmed as source of data. It would be helpful to understand why you picked the databases you did and I would consider adding PubMed as there are definitely studies on IPV pathways in conflict (particularly in LMICs) that didn’t make it into your final list.

I will note your search is relatively old now - typically I’d want to see an article for review with a search about a year old and we are coming up on 2 in February. Given that I think major changes/revisions are needed - you may also consider updating your search.

I would expect to see your full search terms/string in an appendix or annex. I should be able to copy and paste it to replicate your search. Please add.

I would expect that all aspects of the PRISMA checklist are included in your article (https://static1.squarespace.com/static/65b880e13b6ca75573dfe217/t/67ad313f1c80aa5235fce0d0/1739403584136/PRISMA_2020_checklist.pdf) - your methods section does not give the expected level of detail. Please cross check against the checklist and add. For example, how many people did the screening, etc.

Results and Discussion

Overall I think you have a lot of interesting findings - but they don’t map as “mechanisms” to IPV for me as there is so much differences in the populations - military vets, formerly abducted child soliders, conflict-affected populations in both high and low income countries, refugees living in high income countries, etc. I think to make the results meaningful you would need to separate findings by populations in some way. Maybe consider adding a diagram of the mechanisms you found and noting which populations these were identified in. Something to bring your findings together which right now feel a bit dis-jointed.

I am also not sure about your section on perpetration rates - from what I can tell much of that data isn’t from population-based samples so I am concerned about presenting these rates without context. Similarly victimization rates - you are presenting military and non military samples, differing sample techniques, etc. From your title and abstract I wasn’t expecting this much of the paper to spent on these rates - and I think they can be misleading without further stratification and analysis. I would remove these sections and just focus on your mechanisms question.

For mechanisms - as mentioned above I think you need to breakdown your findings more by type of population first. Then in the discussion you can bring together similarities and differences between populations - reflecting on what the evidence actually suggests for these groups and where the gaps are. Also, I think your section on structural and societal contains a lot - consider adding sub-headings (poverty, alcohol use, etc.).

There is also no assessment of the quality of the studies you included or a GRADE assessment of the overall quality of the evidence. This would be needed.

Overall you have a lot there and I encourage you to keep going - but I do think there is more to do before this is ready for publication.

# Reviewer report: Review: Drivers of the association between armed conflict and intimate partner violence: A systematic review — R0/PR3

**DOI:** 10.1017/gmh.2026.10248.pr3

Introduction

• Line 47: The sentence, ‘the complex reasons for the increased prevalence…’ is misleading because it suggests that there is recent scholarship on this topic, yet the sentence only cites an article from 2010. Suggest reframing this sentence or adding in more recent citations

Methodology

• A significant body of literature on this topic can be found on Pubmed, but it is not included as one of the databases. Could the authors share why this was not included? I would recommend redoing the search with this included to see the overlap of potential duplicate articles and to add in any relevant articles only found on PubMed.

• How many people reviewed the titles and abstracts? What was the reliability between multiple reviewers? How was quality of the review assessed?

• To examine mechanisms, quantitative studies typically need to be longitudinal. Was this included as a criteria for inclusion? Or were most studies cross sectional in which variables could either be considered mechanisms or confounders to the associations between conflict and IPV?

• The validation workshop is helpful – could the authors add in more information as to the specific types of stakeholders and why this was focused only on Uganda and not the broader humanitarian system?

• Why would the authors not restrict the literature to research that occurred in LMICs?

• Please include tables of search terms

Results

• The presentation of qualitative studies is strong

• From my understanding of the literature, there are many studies that describe an association between conflict and IPV that are not included in the quantitative results. This may be due to not including PubMed. Additionally some of the quant studies are only correlational of cross-sectional data which limits ability to make inferences of mechanisms.

Discussion

• Given girls aged 15+ are considered to be of reproductive age and adults in their day to day realities, it is substantial limitation of the study to not include these studies. How many were not included because of this reason?

• The discussion points to several possible mechanisms. A diagram that visually illustrates these would be helpful to synthesize the results for the reader.

• The discussion would benefit from including this framework article and position its findings in relation to the model: Murphy, et al. Socioecological Framework for Drivers of Conflict and Postconflict Violence Against Women and Girls

https://journals.sagepub.com/doi/abs/10.1177/10778012221094065

# Editor report: Recommendation: Drivers of the association between armed conflict and intimate partner violence: A systematic review — R0/PR4

**DOI:** 10.1017/gmh.2026.10248.pr4

**Affiliations:** Program on Forced Migration and Health, Columbia University Mailman School of Public Health, United States

We have received comments from two reviewers, both of whom recognize the importance of this topic and the value of synthesizing the existing research. We encourage the authors to carefully review the feedback provided and revise the manuscript accordingly.

In particular, both reviewers recommend expanding the search strategy to ensure broader coverage of the literature, including the addition of PubMed. We suggest incorporating these recommendations as you prepare your revised manuscript.

# Editor report: Decision: Drivers of the association between armed conflict and intimate partner violence: A systematic review — R0/PR5

**DOI:** 10.1017/gmh.2026.10248.pr5

**Affiliations:** Johns Hopkins University Bloomberg School of Public Health, United States

No accompanying comment.

# Reviewer report: Review: Drivers of the association between armed conflict and intimate partner violence: A systematic review — R1/PR7

**DOI:** 10.1017/gmh.2026.10248.pr7

I think you have adequately addressed the major comments. Please do a copy edit of the document as - particularly with the tracked changes - there are some incomplete sentences/editing of some awkward phrasing needed. I think it may be helpful to have a short table with the drivers you identified and then which samples they were seen in to tie your narrative together and make it easier to follow. For your section on refugees and IDPs - it does look like your studies are just for refugee populations (please ignore this comment if incorrect). If so, I would remove the IDP from the title and the “refugee and IDP” terminology. Given the strong overlap between IDP and general conflict affected populations, just focusing on refugee populations in this section also makes more conceptual sense to me.

# Editor report: Recommendation: Drivers of the association between armed conflict and intimate partner violence: A systematic review — R1/PR8

**DOI:** 10.1017/gmh.2026.10248.pr8

**Affiliations:** Program on Forced Migration and Health, Columbia University Mailman School of Public Health, United States

Thank you for submitting your revised manuscript. The reviewer is pleased to note that the majority of their comments have been addressed and has offered a few remaining minor suggestions, which I encourage you to consider.

# Editor report: Decision: Drivers of the association between armed conflict and intimate partner violence: A systematic review — R1/PR9

**DOI:** 10.1017/gmh.2026.10248.pr9

**Affiliations:** Johns Hopkins University Bloomberg School of Public Health, United States

No accompanying comment.

# Editor report: Recommendation: Drivers of the association between armed conflict and intimate partner violence: A systematic review — R2/PR11

**DOI:** 10.1017/gmh.2026.10248.pr11

**Affiliations:** Program on Forced Migration and Health, Columbia University Mailman School of Public Health, United States

No accompanying comment.

# Editor report: Decision: Drivers of the association between armed conflict and intimate partner violence: A systematic review — R2/PR12

**DOI:** 10.1017/gmh.2026.10248.pr12

**Affiliations:** Johns Hopkins University Bloomberg School of Public Health, United States

No accompanying comment.
